# Targeted gene knock-in by CRISPR/Cas ribonucleoproteins in porcine zygotes

**DOI:** 10.1038/srep42458

**Published:** 2017-02-14

**Authors:** Ki-Eun Park, Anne Powell, Shelley E. S. Sandmaier, Chan-Mi Kim, Alan Mileham, David M. Donovan, Bhanu P. Telugu

**Affiliations:** 1 Department of Animal and Avian Sciences, University of Maryland, MD, USA; 2 Animal Bioscience and Biotechnology Laboratory, USDA-ARS, Beltsville, MD, USA; 3 Renovate Biosciences Inc, Reisterstown, MD, USA; 4 Genus plc, DeForest, WI, USA

## Abstract

The domestic pig is an important “dual purpose” animal model for agricultural and biomedical applications. There is an emerging consensus in the biomedical community for the use of large animal models such as pigs to either serve as an alternative, or complement investigations from the mouse. However, the use of pig has not proven popular due to technical difficulties and time required in generating models with desired genetic modifications. In this regard, the ability to directly modify the genome in the zygote and generate edited animals is highly desirable. This report demonstrates for the first time, the generation of gene targeted animals by direct injection of Cas9 ribonucleoprotein complex and short stretches of DNA sequences into porcine zygotes. The Cas9 protein from *Streptococcus pyogenes* was pre-complexed with a single guide RNA targeting downstream of the ubiquitously expressed *COL1A* gene, and co-injected with a single-stranded repair template into porcine zygotes. Using this approach a line of pigs that carry pseudo attP sites within the *COL1A* locus to enable phiC31 integrase mediated introduction of transgenes has been generated. This new route for genome engineering in pigs via zygote injection should greatly enhance applications in both agriculture and biomedicine.

The domestic pig is an important agricultural and biomedical model species. A growing global human population, an increasing demand for animal protein in human diets, and a population that is living longer, necessitate a renewed focus on developing technologies in “dual purpose” animals such as pigs that can serve both agriculture and biomedicine. From a biomedical standpoint, the domestic pig is an ideal “bridge model” to complement, or even serve as an alternative to the mouse or other phylogenetically distant animals to model human disease. The domestic mouse has contributed to the discovery of many of the currently available drugs and treatments. However, a disproportionate number of clinical trials and consequently drug development efforts have met with an unexpected and pre-emptive failure, attributable in part to unreliable data from the mouse, and in some cases to a failure to replicate the symptoms associated with human mutations^1^,^2^,^3^,^4^. The domestic pig is an ideal alternative to bridge this gap, primarily because of the similarity to humans in size, anatomy and physiology, and a longer life expectancy permitting investigations over a prolonged period of time^5^. In addition, the pig has reproductive attributes that make it a suitable livestock model for genetic engineering experiments. The pig is reproductively active throughout the year, is a litter bearing animal carrying multiple piglets in one pregnancy (averaging 14 piglets in domestic crossbreeds), and has a relatively short gestation interval (114 days). That said, the true potential of pig (or other suitable livestock animal models) is only just beginning to be realized with the advent of genome editors.

In pigs and other livestock, gene targeting and transgenesis is typically carried out in somatic cells prior to generating modified animals via somatic cell nuclear transfer (SCNT). Manipulation of somatic cells for targeted insertion of transgenes (knock-in), introduction of subtle changes (point mutations), or ablation of genes by homologous recombinant (HR) based gene targeting has poor efficiencies. Correct recombination events are rare (1 in 10^6^–10^7^ cells), and often monoallelic, and require a second round of targeting for generating homozygous/biallelic modifications. However, the efficiencies can now be improved more than 1000-fold by introducing a double-strand break (DSB) at the target locus^6^ using engineered nucleases such as ZFNs (zinc finger nucleases), TALENs (transcription activator-like effector nucleases), and CRISPR (clustered regulated interspaced short palindromic repeat) and CRISPR-associated (Cas) nuclease system (CRISPR/Cas). Of the three widely used editors, the CRISPR/Cas system is emerging as the tool of choice because of the ease of design, delivery, and relatively high fidelity in engineering DSBs. The CRISPR/Cas system has evolved in archaea and eubacteria as an RNA-based adaptive immunity system to detect and cleave invading viruses and plasmids^7^,^8^. The *Streptococcus pyogenes* (Sp) Type II CRISPR/Cas system has been adapted to successfully modify somatic cells and generate modified pigs^9^,^10^,^11^,^12^,^13^. In these studies, a mammalian codon-optimized Cas9 nuclease along with a synthetic single-guide RNA (sgRNA)^14^ has been used to generate genetically modified pigs^9^,^10^,^11^,^12^,^13^. Although, live offspring have been generated using CRISPRs and SCNT, the difficulties in culturing primary somatic cells long enough to edit, clonally propagate, and screen for correct recombinants, coupled with the challenging task of using the somatic cells for nuclear transfer, makes the technique intractable for an average laboratory. An often overlooked and less often discussed difficulty is targeting certain loci that are within heterochromatin regions of the genome in somatic cells requiring additional rounds of targeting or screening more clonal lines. Direct injection of editors into porcine zygotes may overcome many of these difficulties. A few publications have already successfully demonstrated the generation of bi-allelic knock-out pigs by injection of editors into zygotes^13^,^15^,^16^. A recent report showed the successful introduction of point mutations by microinjection in pig zygotes using zinc finger nucleases^17^. Additionally, gene targeting has been achieved *in vitro* by microinjecting targeting reagents and CRISPR/Cas reagents into porcine zygotes; although high degree of mosaicism has been observed^18^. In the current study, we sought to go a step further and demonstrate that it is possible to knock-in short DNA sequences (or transgenes) into target loci by injection into *in vivo* derived zygotes and to generate live offspring bearing targeted mutations using the CRISPR/Cas system. As a proof of concept, we sought to introduce LoxP and pseudo-attP sites into sites permissive for the subsequent introduction of functional transgenes. We hypothesise that the genome in early zygote will be in an open conformation and thus ideal for targeting most loci with relative ease. Additionally, in porcine and other large animal models, the pronuclei are often not clearly visible in early zygotes. This work avoids the need to inject into the pronucleus (as is often required for zygote injections). Rather, it is only necessary to inject the CRISPRs and targeting oligonucleotides into the cytoplasm in order to generate targeted offspring.

## Results

### Targeted knock-out of alleles by cytoplasmic injection into parthenogenic and *in vitro* fertilized embryos

*In vitro* transcribed SpCas9 and sgRNA targeting *PRNP* (Prion gene) were injected 5 hours (h) following parthenogenetic activation or *in vitro* fertilization of porcine oocytes. SpCas9 is a fusion construct with green fluorescent protein (GFP) and nuclear localization signals (NLS) that allows for tracking the expression of full length Cas9 protein and its translocation into the nucleus of the zygote. As shown in Supplementary Fig. S1, expression of full length GFP fused Cas9 protein can be seen in zygotes with some of the protein translocating into the nucleus of the zygote 24 h following injection. Screening of injected embryos at the blastocyst stage demonstrated successful bi-allelic targeting of the *PRNP* locus as evidenced by large deletions (Supplementary Fig. S1). Injection of *in vitro* transcribed SpCas9 mRNA and a sgRNA targeting a second gene target, *ZBED6*, as well as a combination of guides targeting *PRNP* and *ZBED6*, generated indels causing knock-out of the *ZBED6* gene (Supplementary Fig. S2), or knock-out of both *PRNP* and *ZBED6* genes (Supplementary Fig. S3) respectively, in porcine zygotes. Following the success of targeting a single or a combination of genes by intracytoplasmic injection of porcine zygotes in our preliminary studies, we sought to optimize the concentrations of CRISPR reagents to assess the percentage of gene targeting and developmental rates in porcine embryos following CRISPR injections (Supplementary Fig. S4). Serial 2-fold dilutions of Cas9: sgRNA starting from 100 ng/μl Cas9 and 50 ng/μl sgRNA were injected into porcine zygotes to generate mutations at the target sites. This confirmed a range of concentrations within which CRISPRs can introduce mutations at the target sequences.

### Targeting knock-in of alleles by cytoplasmic injection into parthenogenetic and *in vitro* fertilized embryos

**Figure 1:**
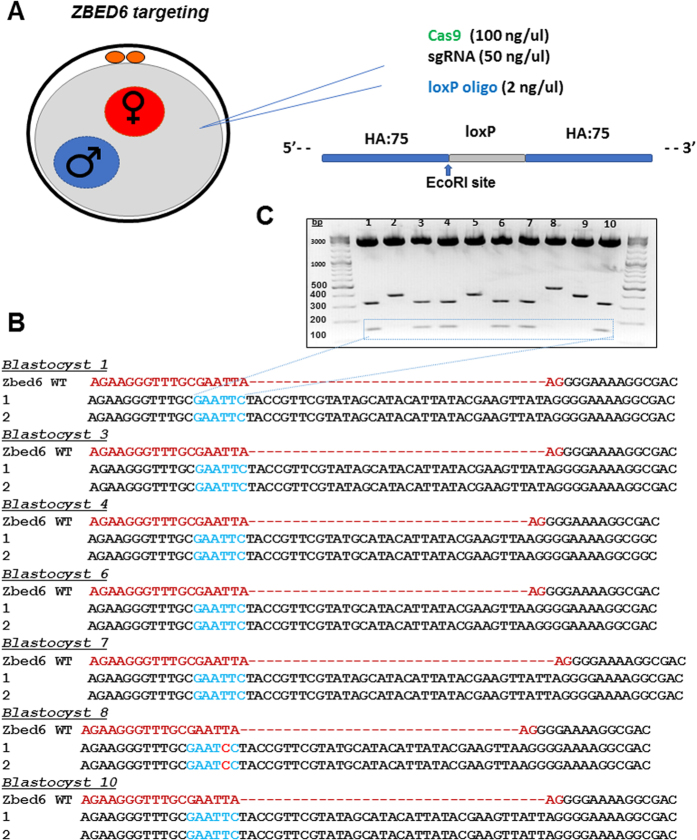
Targeted knock-in of a LoxP site into *ZBED6* allele. **(A)** Schematic of the microinjection of targeting mixture consisting of *in vitro* transcribed Cas9: GFP mRNA, sgRNA targeting *ZBED6* and a single stranded targeting oligo containing 75 bp homology arm (HA) on either side of the CRISPR cut-site and 34 bp LoxP site (184 bp total). One of the nucleotides on the left HA has been modified to facilitate an A: C substitution (arrow) and generation of a novel EcoR1 site following successful targeting. (**B**) Following microinjection, the embryos were cultured to blastocyst stage, lysed, PCR amplified, cloned into PCR2.1 vector (Life Technologies), and plasmid DNA from five bacterial colonies sequenced by Sanger sequencing. Representative colonies that showed knock-in into one of the two alleles were shown (wildtype allele not shown). Sequencing of clones from 10 blastocysts revealed that 7/10 embryos had efficient knock-in of the LoxP site (highlighted in blue), however blastocyst# 8 had mutations in the EcoR1 site. Blastocyst # 9 had no knock-in of LoxP site. (**C**) Agarose gel electrophoresis of EcoR1 digested plasmids from each blastocyst that were confirmed by sequencing. The cloning vector has two EcoR1 sites, and the knock-in alleles will have reconstituted a third EcoR1 site. Consequently, a clone with wild type sequence is expected to release 445 bp insert (Blastocysts# 2,5, and 9), whereas a successful knock-in of EcoR1 site is expected to release 2 fragments of 329 bp and 150 bp (Blastocysts# 1, 3, 4, 6, 7, and 10), in addition to the 3.9 kb vector backbone (top band). Blastocyst # 8 was successfully knocked-in, however, the EcoR1 site was mutated resulting in 2 bands being generated, and a shift in the size of the top band due to knock-in of a truncated (32 bp) LoxP site (477 bp).

Following the initial success with generating knock-outs *in vitro*, we sought to systematically optimize conditions for targeted knock-in of short stretches of DNA sequences as well as long transgene constructs into porcine zygotes. Single stranded DNA (ssDNA) oligonucleotides (hereafter referred to as “oligo”) have proven effective in facilitating homology directed repair (HDR) driven knock-in of short stretches of sequences into porcine zygotes^11^. Here we attempted to knock-in a 34-base pair (bp) LoxP site into the *PRNP* and *ZBED6* genes by co-injection of SpCas9, sgRNA and ssDNA targeting oligo. The targeting oligo were designed with 75 bp of homology arm (HA) on either side of the SpCas9 cut site in each target allele respectively. Additionally, the oligo was designed (with 1 bp modification) to create a new EcoR1 site following HDR knock-in to facilitate screening (Fig. 1A). As shown in Fig. 1B, cloning of amplicons and Sanger sequencing of 5 bacterial clones each from 10 different blastocysts confirmed reconstitution of EcoR1 site in 6/10 blastocysts (Blastocysts# 1, 3, 4, 6, 7, and 10). No knock-in was evident in 3/10 blastocysts (Blastocysts # 2, 5 and 9), whereas a mutated EcoR1 was identified in one of the blastocysts (Blastocyst# 8). Fidelity of the reconstituted EcoR1 site in the knock-in embryos was further investigated by EcoR1restriction digestion (Fig. 1C). The cloning vector (PCR2.1; Life Technologies) has two EcoR1 sites flanking the cloned fragment. As expected, digestion of plasmids bearing wild type sequence (blastocysts # 2, 5 and 9) with EcoR1 resulted in the release of one insert (445 bp; bottom band) from the vector backbone (3.9 kb; top band) when resolved on an agarose gel. However, as anticipated, restriction digestion of representative clones with an EcoR1 site knock-in (from blastocysts# 1, 3, 4, 6, 7, and 10) resulted in release of two fragments (329 bp and 150 bp; bottom bands) from the vector backbone (top band). That said, among the blastocysts with a functional EcoR1 site, only two blastocysts (blastocyst #1 and 3) showed full length (34 bp) LoxP sequence (Fig. 1B), with the remainder embryos showing 1–2 bp insertions or deletions in the introduced LoxP site potentially due to errors in oligo synthesis or DNA repair.

**Figure 2:**
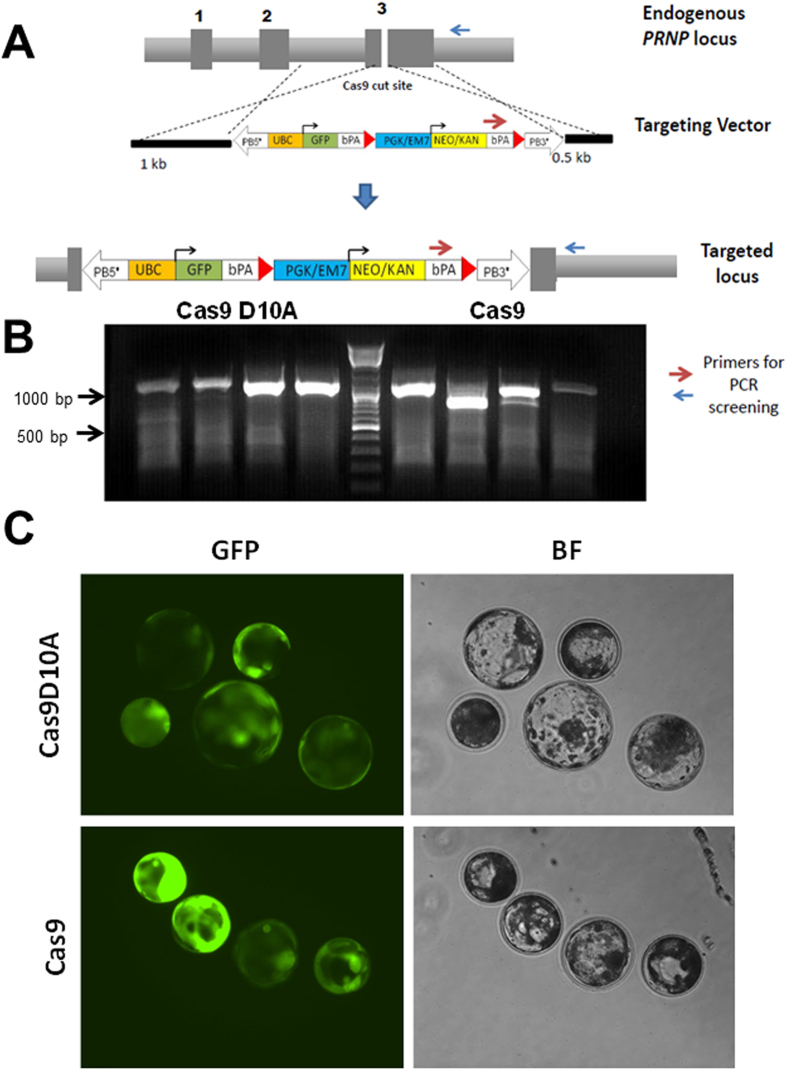
CRISPR-mediated knock-in of GFP transgene into the *PRNP* locus. (**A**) Schematic of the targeting vector showing targeting homology arms (HA) to the pig *PRNP* locus and strategy for knock-in. In the targeting vector, the upper arm is 1000 bp and the lower arm 500 bp in length. The linearized targeting vector, Cas9 mRNA and sgRNA targeting *PRNP* were co-injected into the cytoplasm of porcine zygotes. As shown in the schematic, Cas9 induces double strand break in exon 3 of *PRNP* gene. The cut DNA is then repaired by HDR using the targeting vector, resulting in the targeted allele. The use of Cas9D10A (nickase) introduced a single stranded nick as compared to DSB by Cas9, triggering HDR mediated knock-in into the *PRNP* locus. Dark grey boxes represent exons of the *PRNP* gene. (**B**) PCR amplification using primers one within the targeting vector and another outside of the targeting vector produced specific bands of 1050 bp confirming targeting to the intended locus. In the figure, the 1000 bp and 500 bp markers with corresponding bright bands on the ladder are shown as a reference. (**C**) Representative knock-in embryos that have developed to the blastocyst stage showed varied GFP expression in both Cas9D10A and Cas9 injections.

The ability to site-specifically knock-in transgenes into a specific locus that eliminates the possibility of insertional mutagenesis, positional variegation, expression of transgenes and viability of the resulting animal is highly desired for generating transgenic animals. To achieve this objective, we targeted two constitutively expressing loci (*PRNP* and *COL1A)*. Injection of a double stranded *GFP* transgene targeting the *PRNP* locus with 1000 bp and 500 bp of homology on either side of the CRISPR cut site, along with the CRISPR reagents (Cas9 nuclease or Cas9D10A nickase and sgRNA complex) into the embryos led to the targeted knock-in of the transgene into the *PRNP* locus (Fig. 2 and Supplementary Fig. S5). As a control, only Cas9:GFP RNA or vector only injections were performed. Even though, residual expression of GFP was noticed in plasmid only controls, site-specific integration of the *GFP* transgene and persistent expression of *GFP* transgene was noticed only in embryos derived from injection of targeting vector and Cas9 or Cas9D10 nickase RNA (Fig. 2B,C and Supplementary Fig. S5). That said, full length amplicons were identified in the nickase injections, whereas truncations in the 3′ end around the cut-site was identified in Cas9 nuclease injections. This is because, the *PRNP* targeted sequence has intact guide RNA sequence following knock-in of *GFP* transgene that resulted in subsequent targeting by Cas9 resulting in cleavage and truncation.

**Figure 3:**
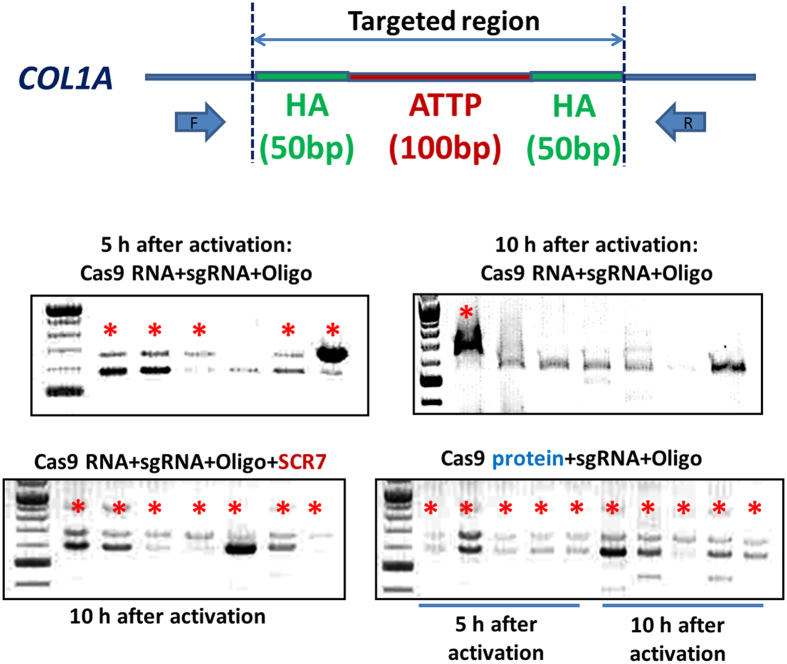
Knock-in of pseudo attP sites downstream of the ubiquitously expressed *COL1A* locus. Targeted knock-in of two pseudo attP sites (50 bp each; 100 nt total) downstream of the polyA site of the *COL1A* locus. Injection of a targeting complex containing Cas9 mRNA or protein, in the presence or absence of DNA ligase IV inhibitor (SCR7) at two different time points has been investigated. Injected zygotes in batches of 25 were cultured for 6 days and the blastocysts were screened with primers spanning the targeted region. As was shown here, the use of Cas9 protein was effective in introgressing pseudo attP sites into the *COL1A* locus as evident from 100 bp shift in the product size (two 50 bp attP sites) denoted by asterix. In the figure, the bottom band represents the wildtype allele. The final concentrations of the reagents are: Cas9 RNA: 25 ng/μl; Cas9 protein: 25 ng/μl; sgRNA: 12.5 ng/μl; Oligo: 50 ng/μl; and SCR7: 10 μM.

In a second experiment, we targeted downstream of the polyA site of the *COL1A* locus to knock-in two pseudo attP sites (the recombination sites for commercially available phiC31 integrase). The phiC31 integrase recognizes pseudo attP sites and can mediate recombination and integration of engineered transgenes into the *COL1A* locus. A ssDNA oligo with 50 bp of homology to a target site downstream of the *COL1A* polyA site was used to introduce two pseudo attP sites (50 bp each; two pseudo attP sites total 100 bp). The pseudo attP sites were based on the published report^19^,^20^. In order to further optimize conditions and facilitate knock-in, we injected the targeting oligo with either *in vitro* transcribed Cas9: GFP mRNA or the Cas9 protein. Additionally, we tested the use of SCR7 (a specific inhibitor of DNA ligase IV)^21^,^22^ in biasing the repair pathway towards HDR as opposed to the non-homologous end joining (NHEJ) repair pathway. As shown in Fig. 3, the use of Cas9 protein proved to be effective in knocking-in pseudo attP sites into the target locus at two different time points following activation of the embryos. As shown in Fig. 3, injection of the targeting oligos and CRISPR RNA at 5 h after activation resulted in reliable knock-in of attP sites. However, difference in the intensity of wild type (WT) and knock-in banding in Cas9 RNA injections was evident likely suggesting mosaicism. Injection of Cas9 RNA at a later time point (10 h) primarily resulted in ablation, while the use of SCR7 inhibited NHEJ and resulting ablation events, but resulted in recombination of alleles and higher order banding. Injection of precomplexed Cas9 protein and sgRNA ribonucleoproteins (RNP) along with targeting oligos 5 h after activation resulted in highly reliable knock-in, whereas injection at later time points (10 h) clearly resulted in 3–4 distinct alleles. Based on this preliminary evidence, Cas9 RNP was chosen for injection 5 h after activation as a means for achieving reliable knock-in of short sequences and generation of targeted animals.

### Generation of attP knock-in pigs by direct injection of CRISPR reagents into porcine zygotes

**Figure 4:**
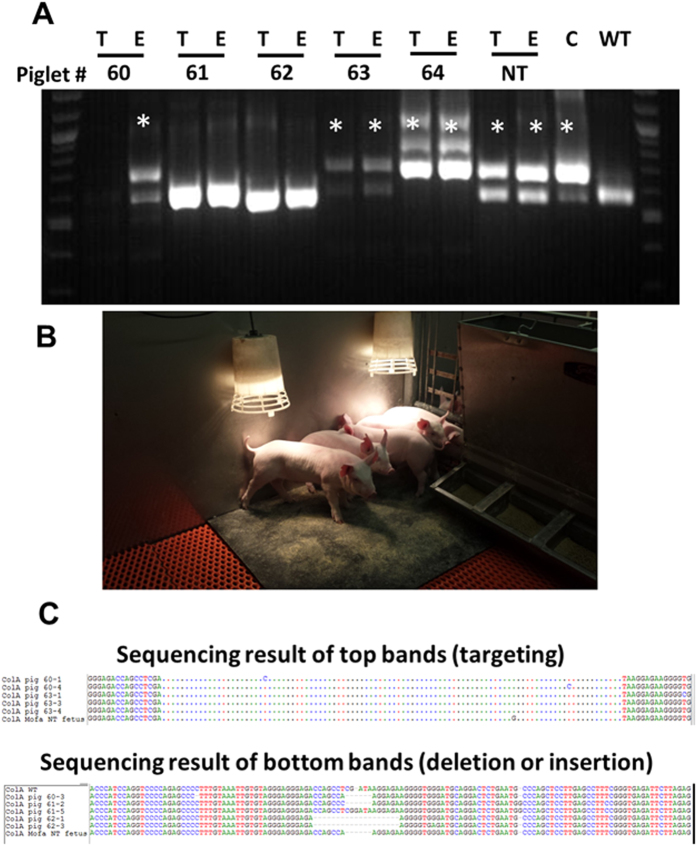
Piglets generated by targeted knock-in of pseudo attP sites into the *COL1A* locus using a Cas9 ribonucleoprotein complex. Injection of targeting mixture consisting of Cas9 protein complexed with sgRNA and a targeting oligo into *in vivo* matured, *in vitro* fertilized embryos has generated a healthy litter. Genotyping of genomic DNA via PCR from tail (T) or ear (E) samples showed that 3 of the 5 piglets carried targeted knock-in of the pseudo attP sites (denoted by asterix). Control genomic DNA from SCNT derived fetus (NT), targeted fibroblasts (C), and wildtype (WT) pigs is shown for comparison. (**B**) Photograph of the litter generated from embryo transfer. (**C**) Sanger sequencing confirmed the knock-in of 100 bp of pseudo attP sites (Piglet # s 60 and 63). Interestingly, animals that did not undergo targeted knock-in via HDR, had indels at the cut site, diagnostic of NHEJ repair (Piglets # 60 second allele; 61 and 62).

Following *in vitro* validation, we performed embryo transfers to generate animals with knock-in of a LoxP site into the *PRNP* or *ZBED6* loci, or pseudo attP sites downstream of the *COL1A* locus. The summary of embryo transfer experiments is shown in Table 1. In initial trials, the use of *in vitro* fertilized embryos and zygotic injections proved to be unsuccessful in generating edited animals. However, using *in vivo* matured oocytes that were fertilized *in vitro*, or the use of *in vivo* derived zygotes resulted in successful pregnancies. As shown in Fig. 4 and Supplementary Figs S6 and S7, these experiments resulted in litters ranging from 3 to 7 piglets, and observed knock-in efficiencies between 0% and 66% of the resulting fetuses carrying a targeted knock-in. Following the birth of the first batch of live animals, pregnancies that have progressed further and that ensure a successful pregnancy outcome were terminated at day 60 of pregnancy. At this stage, the piglets have not started respiration, and the fetuses will not have to be euthanized, but can be collected to assess targeting efficiencies. Among the live offspring that have been generated, evidence for any mosaicism was investigated at 6 months of age. As shown in Supplementary Fig. S8, no evidence of mosaicism (only 2 alleles noted) was evident in tissues from the 3- germ layers that were investigated at the time of euthanasia at 6 months of age. Additionally, in the offspring, no evidence for off-targeting was found in the top two exonic and non-exonic off-target regions (Supplementary Fig. S9). To test the functionality of the pseudo attP sites, a *GFP* donor vector containing a complementary attB site was co-transfected with a plasmid expressing mammalian codon optimized phiC31 integrase. The resulting cells were selected for 2 weeks with G418, the GFP positive cells flow sorted and screened for targeted knock-in. As shown in Supplementary Fig. S10, only cells knock-in for pseudo attP sites in the *COL1A* locus showed site-specific integration of the GFP transgene and not the control wildtype cells.

## Discussion

The importance of the domestic pig as a model for agricultural and biomedical applications is well documented. From a biomedical standpoint, arguably the pig (and other livestock species) is better suited for investigating diabetes, cardiovascular and metabolic disorders than the mouse or other phylogenetically distant animal models. However, the widespread use of pigs in biomedical studies has not been realized despite a broad consensus on the utility of pig models within the biomedical community. Apart from considerations such as costs associated with housing and maintaining large animals and long generation interval, the time and effort it takes in generating genetically modified pigs carrying targeted modifications is a significant limitation to the use of pigs in biomedicine. Before the adoption of genome editing, achieving sophisticated modifications such as targeted gene knock-ins, knock-outs, and introducing point mutations was very inefficient, laborious and often unpredictable. Being able to efficiently generate genetically modified pigs in a single generation will greatly enable the use of pigs in generating biomedical models, and direct injections into the zygotes of pigs will be an important contribution.

The advent of genome editors has been a game changer for genetic engineering of pigs and other livestock, which have not witnessed a major breakthrough since cloning or nuclear transfer two decades ago^23^,^24^. The CRISPR/Cas system is ushering in a revolution in this field. The CRISPRs are easy to engineer for an average laboratory as they require the use of just two reagents, Cas9 mRNA or a commercially available Cas9 protein preparation, and a chimeric single guide RNA that can be designed and *in vitro* transcribed or purchased commercially. The CRISPRs engineer double strand breaks at the target site with high reliability. This double strand break is a precursor for targeted modifications, including disruption of the open reading frame and generation of knock-out animals, introduction of short or long stretches of sequences, and more importantly point mutations. The main objective of this study is to showcase the utility of these tools in the context of zygotes and the advantages they offer, such as being able to engineer targeted mutations directly in the embryos and generate edited animals in one generation. In this study, the introduction of DSBs coupled with the use of ssDNA oligos has engineered targeted mutations in 33–60% among viable piglets. An added advantage to performing gene targeting in zygotes is the elimination of the need to engineer mutations in somatic cells and subsequent SCNT. This work relieves the difficulties in achieving targeted mutations in somatic cells, especially those genomic regions that are highly refractory to modification (regions that are transcriptionally silent in somatic cells and potentially within heterochromatic regions (not shown)). SCNT is a challenging technology beyond the capability of most laboratories. That said the use of zygotes also has its own set of limitations. As shown in Table 1, our use of *in vitro* fertilized embryos failed to establish a successful pregnancy even though we could show that IVF embryos had undergone gene targeting *in vitro*. In pigs, *in vitro* matured oocytes fail to mount an effective block to polyspermy, resulting in fertilization from more than a single sperm, and consequently a failure to generate viable embryos and establish pregnancies^25^,^26^. A second concern is zona hardening, where the expanded embryos fail to hatch from the hardened zona and establish pregnancy. The use of *in vivo* matured oocytes that have been *in vitro* fertilized and subsequently injected with the CRISPRs have resulted in successful pregnancies (Fig. 4). Likewise, *in vivo* fertilized embryos have established pregnancies; however, the efficiency of targeting diminished with the age of the embryos. One concern with the use of *in vivo* fertilized embryos for HDR is the difficulty in accurately and reliably obtaining newly formed zygotes, as the ovulation takes place over a 4 h window of time in pigs. In conclusion, HDR rates were highest when we used *in vivo* matured and *in vitro* fertilized oocytes with timed injections. Another potential drawback for the use of *in vivo* matured oocytes is that a larger number of animals required for an experiment as compared to SCNT. However, being able to achieve homozygous targeting, potentially offsets this disadvantage. Focus of future investigations will be to further optimize the timing of injections after zygotes formation, the concentration and molecular nature of the targeting vectors to result in high frequencies of homozygous targeting, and further refining the process. We believe that this work represents an important step forward in achieving routine targeted genetic modifications in pigs.

## Materials and Methods

### Production of Cas9 mRNA and sg RNA

Expression plasmid for Cas9 nuclease (pMJ920) was a gift from Jennifer Doudna (Addgene plasmid # 42234)^27^, and Cas9_D10A nickase was a gift from George Church (Addgene plasmid # 41816)^28^. The SpCas9 and Cas9D10A plasmids were gel purified and used as the template for *in vitro* transcription using mMESSAGE T7 ULTRA kit (Life Technologies). Targeting guide RNAs were designed based on the software available from MIT (http://www.genome-engineering.org/crispr/). Two complementary sgRNA oligo DNAs (19–22 nucleotides in length depending on the guide sequence) were synthesized by IDT (IDT DNA technologies), annealed to form double-strand DNA and cloned into a Bsa1 restriction enzyme digested in-house T7 promoter driven vector. The cloned fragments were DNA sequenced to confirm their fidelity and *in vitro* transcribed using MEGA shortscript T7 kit (Life Technologies) to generate chimeric single guide (sg) RNAs. *In vitro* transcribed Cas9, Cas9_D10A mRNA and the sgRNA were purified with MEGAclear kit (Life Technologies) and eluted in RNase-free water for embryo injections.

### Assembly of targeting vector

A gene targeting vector was generated consisting of 1000 bp and 500 bp upper and lower arms homologous to the targeted cut site bordering exon-3 of *PRNP* gene. Using primers bearing unique restriction sites for AscI, and XhoI, 1000 bp of sequence of upper arm was PCR amplified, restriction enzyme digested, gel purified and ligated into a similarly cut GFP expressing *piggyBac* vector (Sanger Institute)^29^. Likewise, using primers consisting of unique BsiWI and MluI sites, 1000 bp of sequence downstream of the cut site were amplified and cloned into the same GFP *piggyBac* vector. Standard molecular techniques were used to select for positive clones and all clones were confirmed with DNA sequence analysis. The AscI, HpaI fragment carrying 1000 bp upper arm and 500 bp lower homology arm was used for microinjection and gene targeting.

### *In vitro* maturation, *in vitro* fertilization and embryo culture

All chemicals were obtained from Sigma Chemical Company (St. Louis, MO) unless stated otherwise.

Cumulus oocyte complexes (COCs) from abattoir sows were purchased from ART Inc. (Madison, WI) or DeSoto Biosciences Inc. (Seymour, TN) and shipped to the lab overnight in commercial Maturation medium #1(provided by ART or DeSoto) at 38.5 °C. Twenty-four hours (h) after being placed in the Maturation medium #1, 50 to 75 COCs were placed in commercial Maturation medium #2 (provided by ART or DeSoto) and cultured for an additional 20 h at 38.5 °C and 5% CO_2_ in air, 100% humidity. COCs were vortexed in 0.1% hyaluronidase in HEPES-buffered medium containing 0.01% PVA for 4 minutes to remove the cumulus cells following maturation. Groups of 30–35 mature, denuded oocytes were placed in 100 μl of a modified Tris-buffered medium (mTBM) and fertilized according to established protocol^30^ using fresh extended boar semen. One ml of extended semen was mixed with Dulbecco’s Phosphate Buffered Saline (DPBS) containing 1 mg/ml BSA to a final volume of 10 ml and centrifuged at 1000 × g, 25 °C for four minutes, and spermatozoa were washed in DPBS three times. After the final wash, spermatozoa were re-suspended in mTBM medium and added to oocytes at a final concentration of 5 × 10^5^ spermatozoa /ml, and co-incubated for 5 h at 38.5 °C and 5% CO_2_. Presumptive zygotes were used for microinjection and subsequently placed in Porcine Zygote Medium 3 (PZM3) supplemented with 3 mg/ml fatty acid-free BSA^31^ and cultured at 38.5 °C, 5% CO_2_, 5% O_2_ and 100% humidity.

### Parthenogenetic activation

Cumulus-oocyte complexes (COCs) following culture and maturation in Maturation medium II for 20 hours were electro-activated for generation of parthenogenetic embryos. The oocytes were placed in activation medium (300 mM Mannitol, 0.1 mM CaCl_2_, 0.1 mM MgSO_4_, 0.5 mM Hepes, 0.01% BSA) between two platinum electrodes of an Electrocell manipulator (BTX, San Diego, CA) and activated by two DC pulses of 1.2 kV/cm for 30 μs. After 2 minutes of equilibration in activation medium, the embryos were transferred into PZM3 medium and cultured for 7 days. The resulting blastocysts were screened for targeted knock-ins. Parthenogenetic embryos were used for Cas9 and sgRNA titration experiments and for optimization of methodologies for targeting of *COL1A* locus (Fig. 3 and Supplementary Fig. S4).

### Collection of *in vivo* matured oocytes and zygotes for production of gene-targeted pigs via zygote injection with spCas9 protein/sgRNA/ssDNA

All experiments involving live animals were performed in accordance with the approved guidelines of the Beltsville ARS Institutional Animal Care and Use Committee (IACUC). All experimental protocols involving live animals were approved by the IACUC committee. Pubertal gilts were estrus synchronized using Alternogest (Regumate or Matrix, Merck, PA), and checked daily for the onset of estrus (Day 0)^15^. Gilts displaying estrus were inseminated with extended boar semen (Progenes, SD). Zygotes were recovered surgically 24 h later by mid-ventral laparotomy. Briefly, gilts were anesthetized with an intravenous injection of telazol, ketamine, xylazine mixture, and anesthesia was maintained under 5% isofluorane inhalation. The reproductive tract was exposed and the oviducts were flushed with 25 ml of Hepes-buffered medium. The recovered medium was examined under a dissecting microscope to collect the embryos. All recovered *in vivo* zygotes were immediately placed into a drop of TL-Hepes medium and used for microinjection.

### Microinjection of embryos

*In vitro* fertilized, parthenogenetic, or *in vivo* fertilized embryos were microinjected with a mixture of Cas9 mRNA or protein, target sgRNA mixture alongside targeting plasmid or ssDNA using a FemtoJet microinjector (Eppendorf; Hamburg, Germany). The micro-injected parthenogenetic embryos were cultured to blastocyst stage in PZM3 medium for 144 h at 38.5 °C, 5% CO_2_, 5% O_2_ and 100% humidity for screening. The Cas9 protein is purchased from PNA Bio (Newbury Park, CA) and precomplexed as per manufacturers’ recommendations. Briefly 3 μl of 250 ng/μl Cas9 protein is mixed with 3 μl of 250 ng/μl sgRNA and incubated for 10 minutes at room temperature. The resulting Cas9 ribonucleoprotein complex mixture is diluted by 10 fold to a final concentration of 25 ng/μl for microinjection. The *in vitro* fertilized or *in vivo* collected zygotes were subjected to cytoplasmic microinjection with the spCas9 protein/sgRNA/ssDNA mixture as described above. After microinjection, the injected zygotes were *in vitro* cultured for two days and 30–40 four to eight cell stage microinjected embryos were transferred surgically into one oviduct of synchronized recipients.

### Genotyping of embryos, fetuses and edited animals

*In vitro* cultured single blastocysts cultured for 144 h were washed three times with PBS-PVA (pH 7.4) medium and transferred into 9 μl of blastocyst lysis buffer (50 mM KCl, 1.5 mM MgCl2, 10 mM Tris pH 8.0, 0.5% NP-40, 0.5% Tween-20 and 100 μg/ml proteinase K) and incubated for 1 h at 65 °C. The digestion was terminated by heating the mixture at 95 °C for 10 min, and 2 μl of supernatant used as a PCR template. Tissue biopsies (ear notch and tail dock) from fetuses and offspring were digested in a tissue lysis buffer (50 mM Tris pH 8.0, 0.1 M NaCl, 20 mM EDTA, 1% SDS, 50 μg/ml RNase A, 100 μg/ml proteinase K) overnight at 65 °C. Following overnight digest, genomic DNA was extracted using phenol-chloroform, and recovered by resuspension in 100 μl of 10 mM Tris- HCl, pH 7.4 buffer following ethanol precipitation. Genomic DNA from embryos above or purified genomic DNA from tissues was amplified using PCR, cloned into PCR2.1 vectors (Life Technologies) and transformed into Ε. coli DH5-α maximum competent cell (Life Technologies). Ten colonies were picked, cultured, plasmid DNA extracted, and sequenced (Macrogen). Sequences were aligned by Bio-Edit software for comparison with wild-type alleles. Primers used in the manuscript were shown in Supplementary Fig. S11.

## Additional Information

**How to cite this article**: Park, K.-E. *et al*. Targeted gene knock-in by CRISPR/Cas ribonucleoproteins in porcine zygotes. *Sci. Rep.* **7**, 42458; doi: 10.1038/srep42458 (2017).

**Publisher's note:** Springer Nature remains neutral with regard to jurisdictional claims in published maps and institutional affiliations.

## Supplementary Material

Supplementary Information

## Figures and Tables

**Table 1:** Summary of experiments and embryo transfer outcomes.

Experiment	Pregnancy outcome	# Embryos transferred	# of offspring/fetuses	% of transferred embryos that generated fetuses/ offspring	% knock-in	Type of fertilization	Time (h) embryos transferred after injection
1. *PRNP*	Not Pregnant	100	NA	NA	NA	IVF	15
2. *PRNP*	Not Pregnant	90	NA	NA	NA	IVF	15
3. *ZBED6*	Not Pregnant	68	NA	NA	NA	IVF	48
*4a. *COL1A*	Not pregnant	34	NA	NA	NA	*In vivo*	36
*4a. *COL1A*	Pregnant	32	3	9.40%	0	*In vivo*	36
*4b. *COL1A*	Pregnant	54	5	9.30%	60%	IVF	36
*5. *COL1A*	Pregnant	78	7	8.90%	57%	IVF	36
*6. *COL1A*	Pregnant	42	3	7.10%	33%	*In vivo*	36

^*^All zygotes are *in vivo* matured. All transfers into pre-ovulatory recipients except *ZBED6*.
