# Supplementary material for: Targeted gene knock-in by CRISPR/Cas ribonucleoproteins in porcine zygotes
Source: Sci Rep. 2017 Feb 14;7:42458. doi: 10.1038/srep42458 (PMC5307959; doi:10.1038/srep42458)
Supplement: Supplementary Information [file srep42458-s1.pdf]

Targeted gene knock-in by CRISPR/Cas ribonucleoproteins in porcine zygotes

Ki-Eun Park[1,2,3], Anne Powell[2], Shelley E.S. Sandmaier[1,2], Chan-Mi Kim[1,2], Alan Mileham[4], David M. Donovan[2], and Bhanu P. Telugu[1,2,3,*]

[1] University of Maryland, Animal and Avian Sciences, College Park, 20742, USA

[2] USDA-ARS, Animal Bioscience and Biotechnology Laboratory, Beltsville, 20705, USA

[3] Renovate Biosciences Inc, Reisterstown, 21136, USA

[4] Genus plc, DeForest, 53532, USA

[*]Corresponding author (btelugu@umd.edu)

Supplementary Fig. S1. Ablation of *PRNP* in pig embryos. A) Schematic of Cas9:GFP and sgRNA expression plasmids. B) Schematic representing the *PRNP* sequence recognized by the guide RNA and targeted by Cas9 complex. C) Schematic showing an *in vitro* fertilized zygote injected with Cas9 targeting mixture. On the right embryos showing Cas9:GFP expression and localization to the nucleus. D) The PCR amplicons were cloned into PCR2.1 cloning vector and 5 bacterial clones were sequenced to confirm mono-allelic or bi-allelic targeting of the *PRNP* locus.

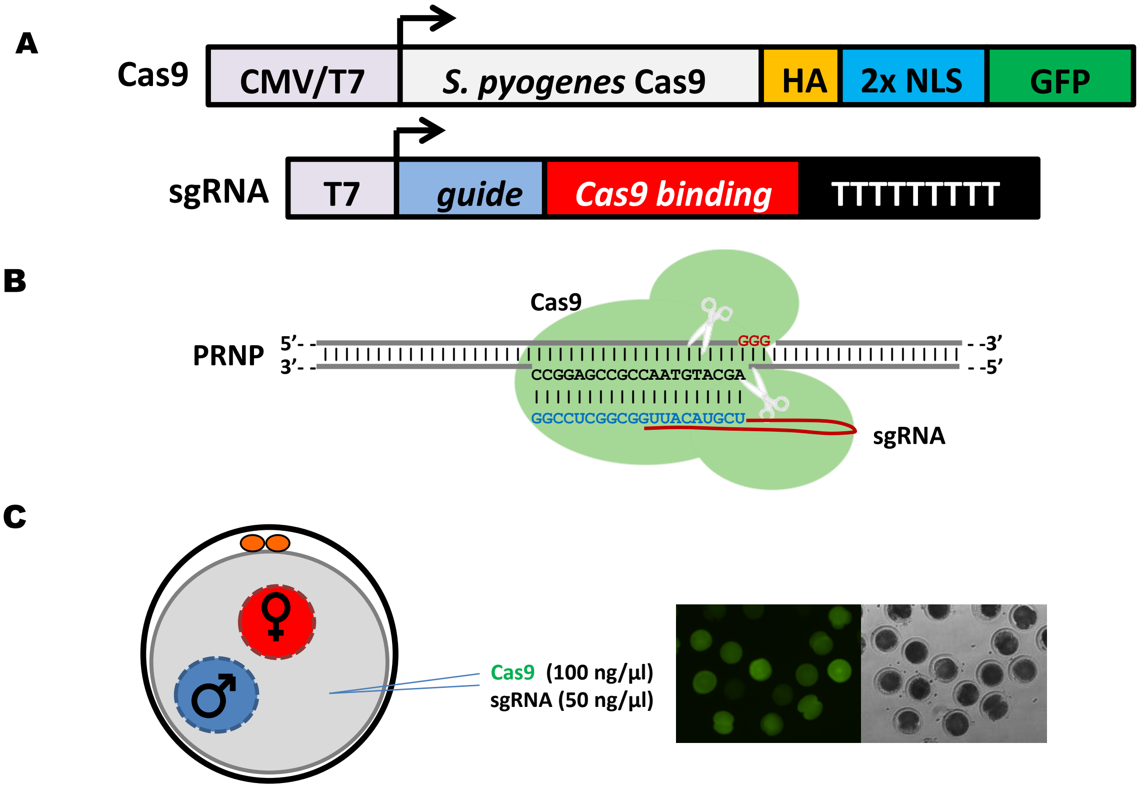

D

```
Blastocyst 1
PRNP     GGTAGGGGGCCTCGGCGGTTACATGCTGGGGAGTGCCATGAGCAGACCCCTGATACACTTT
1        GGTAGGGGGCCTCGGCG----------GGGGAGTGCCATGAGCAGACCCCTGATACACTTT
2        GGTAGGGGGCCTCGGCG----------GGGGAGTGCCATGAGCAGACCCCTGATACACTTT
3        GGTAGGGGGCCTCGGCG----------GGGGAGTGCCATGAGCAGACCCCTGATACACTTT
Blastocyst 2
PRNP     GGTAGGGGGCCTCGGCGGTTACATGCTGGGGAGTGCCATGAGCAGACCCCTGATACACTTT
1        GGTAGGGGGCCTCG----------GCTGGGGAGTGCCATGAGCAGACCCCTGATACACTTT
2        GGTAGGGGGCCTCG----------GCTGGGGAGTGCCATGAGCAGACCCCTGATACACTTT
Blastocyst 3
PRNP     GGTAGGGGGCCTCGGCGGTTACAT-GCTGGGGAGTGCCATGAGCAGACCCCTGATACACTTT
1        GGTAGGGGGCCTCGGCGGTTACATTGCTGGGGAGTGCCATGAGCAGACCCCTGATACACTTT
2        GGTAGGGGGCCTCGGCGGTTACATTGCTGGGGAGTGCCATGAGCAGACCCCTGATACACTTT
Blastocyst 4
PRNP     CCTCGGCGGTTACATGCTGGGGAGTGCCATGAGCAGACCCCTGATACACTTTGGCAGTGACT
1        CCTCGGCGGTTA------------------------------------------CAGTGACT
2        CCTCGGCGGTTA------------------------------------------CAGTGACT
3        CCTCGGCGGTTA------------------------------------------CAGTGACT
```

Supplementary Fig.S2. Ablation of *ZBED6* in pig embryos. A) Schematic representing the *ZBED6* sequence recognized by the guide RNA and targeted by Cas9 complex. B) Schematic showing an *in vitro* fertilized embryo injected with Cas9 targeting mixture. On the right, embryos showing Cas9:GFP expression and localization to the nucleus. C) The PCR amplicons were cloned into PCR2.1 cloning vector and 5 bacterial clones were sequenced to confirm mono-allelic or bi-allelic targeting of the *ZBED6* locus.

A

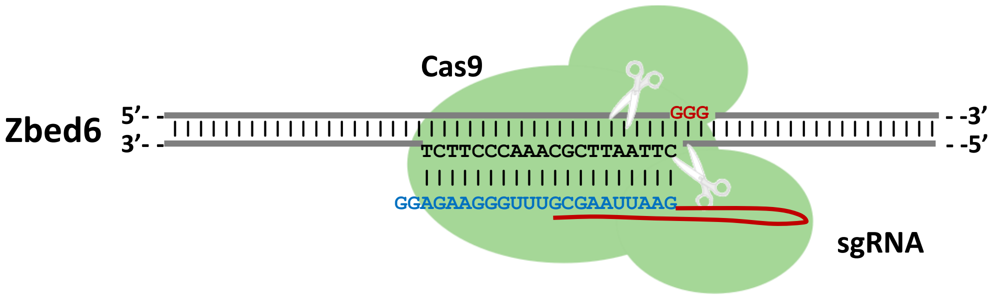

B

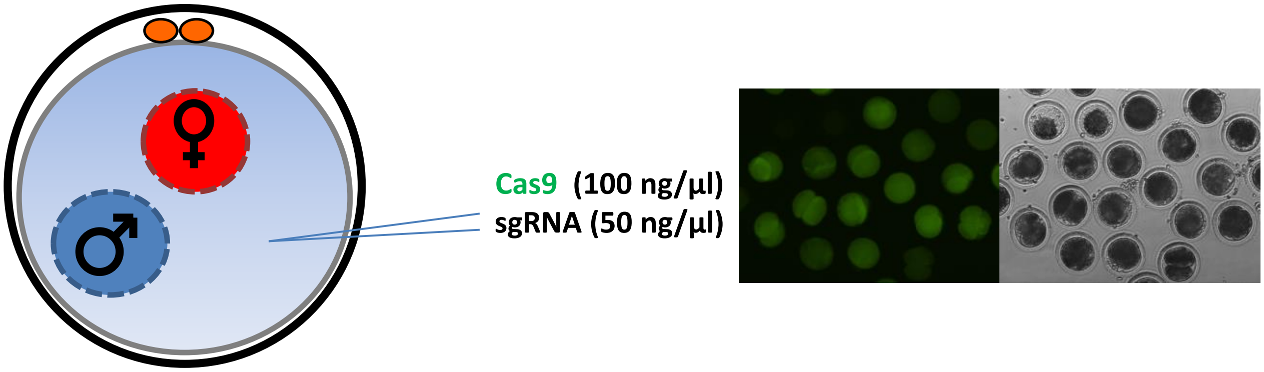

C

```
Blastocyst 1
Zbed6      GAGGCAAAATTGCCTGCCAAAAAGAAAAGAAAGAAGGGTTTGCGAATTAAGGGGAAAAGGCGACGAAA
1          GAGGCAAA--------------------------------------------------GGCGACGAAA
2          GAGGCAAA--------------------------------------------------GGCGACGAAA

Blastocyst 2
Zbed6      GAGGCAAAATTGCCTGCCAAAAAGAAAAGAAAGAAGGGTTTGCGAATT----AAGGGGAAAAGGCGAC
1          GAGGCAAAATTGCCTGCCAAAAAGAAAAGAAAGAAGGGTTTGCGAAGGGGAAAAGGGGAAAAGGCGAC
2          GAGGCAAAATTGCCTGCCAAAAAGAAAAGAAAGAAGGGTTTGCGAAGGGGAAAAGGGGAAAAGGCGAC

Blastocyst 3
Zbed6      GAGGCAAAATTGCCTGCCAAAAAGAAAAGAAAGAAGGGTTTGCGAATTAAGGGGAAAAGGCGACGAAA
1          GAGGCAAA--------------------------------------------------GGCGACGAAA
2          GAGGCAAAATTGCCTGCCAAAAAGAAAAGAAAGAAGGGTTTGCGAA--AAGGGGAAAAGGCGACGAAA
3          GAGGCAAA--------------------------------------------------GGCGACGAAA

Blastocyst 4
Zbed6      GAGGCAAAATTGCCTGCCAAAAAGAAAAGAAAGAAGGGTTTGCGAATTAAGGGGAAAAGGCGACGAAA
1          GAGGCAAAATTGCCTGCCAAAAAGAAAAGAAAGAAGGGTTTGCGAAT-AAGGGGAAAAGGCGACGAAA
2          GAGGCAAAATTGCCTGCCAAAAAGAAAAGAAAGAAGGGTTTGCGAAT-AAGGGGAAAAGGCGACGAAA
```

Supplementary Fig.S3. Ablation of *PRNP* and *ZBED6* double knock-out in pig embryos. A) Expression of full length Cas9:GFP expression. B) The PCR amplicons were cloned into PCR2.1 cloning vector and 5 bacterial clones were sequenced to confirm mono-allelic or bi-allelic targeting of the *PRNP* and *ZBED6* loci.

A

GFP **BF**

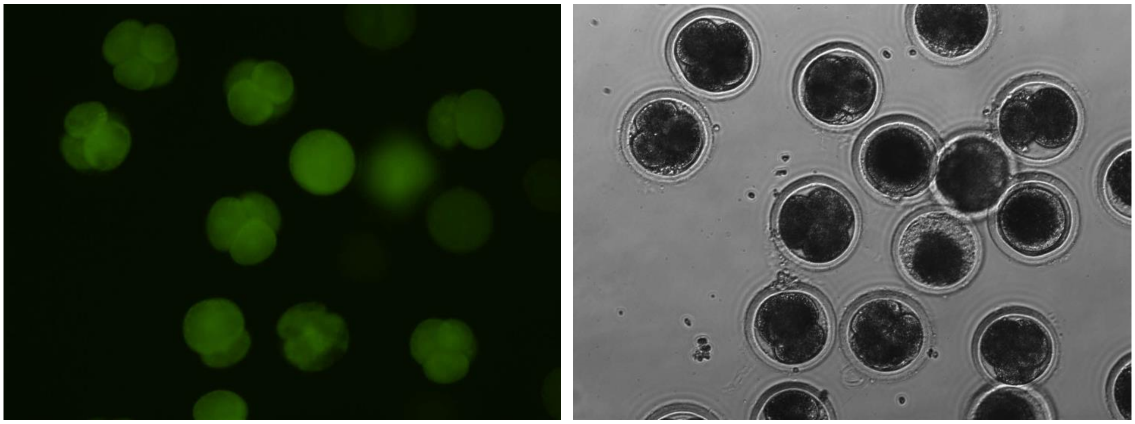

Cas9 (100 ng/ul) +
***PRNP* + *ZBED6* sgRNA (50 ng/ul each)**

B

```
Deletion of PRNP
PRNP          TGGGGCAGTGGTAGGGGGCCTCGGCGGTTACATGCTGGGGAGTGCCATGAGCAGACCCCTGATACAC
1-1           TGGGGCAGTGGTAGGGGGCCTCGGC----------TGGGGAGTGCCATGAGCAGACCCCTGATACAC
2-1           TGGGGCAGTGGTGGGGGGCCTCGGC----------TGGGGAGTGCCATGAGCAGACCCCTGATACAC
2-2           TGGGGCAGTGGTAGGGGGCCTCGGC----------TGGGGAGTGCCATGAGCAGACCCCTGATACAC
3-1           TGGGGCAGTGGTAGGGGGCCTCGGC----------TGGGGAGTGCCATGAGCAGACCCCTGATACAC
3-2           TGGGGCAGTGGTAGGGGGCCTCGGC----------TGGGGAGTGCCATGAGCAGACCCCTGATACAC
4-1           TGGGGCAGTGGTAGGGGGCCTCGGC----------TGGGGAGTGCCATGAGCAGACCCCTGATACAT
4-2           TGGGGCAGTGGTAGGGGGCCTCGGC----------TGGGGAGTGCCGTGAGCAGACCCCTGATACAC
Deletion of Zbed6
Zbed6         GAGGCAAAATTGCCTGCCAAAAAGAAAAGAAAGAAGGGTTTGCGAATTAAGGGGAAAAGGCGACGAAA
1-1           GAGGCA--------------------------------------------------AAGGCGACGAAA
1-2           GAGGCA--------------------------------------------------AAGGCGACGAAA
1-3           GAGGCA--------------------------------------------------AAGGCGACGAAA
2-1           GAGG----------------------------------------------------AAGGCGACGAAA
2-2           GAGGCAAA--------------------------------------------------GGCGACGAAA
2-3           GAGGCAAA--------------------------------------------------GGCGACGAAA
3-1           GAGGCA--------------------------------------------------AAGGCGACGAAA
3-2           GAGGCA--------------------------------------------------AAGGCGACGAAA
3-3           GAGGCA--------------------------------------------------AAGGCGACGAAA
4-1           GAGGCA--------------------------------------------------AAGGCGACGAAA
4-2           GAGGCA--------------------------------------------------AAGGCGACGAAA
4-3           GAGGCA--------------------------------------------------AAGGCGACGAAA
```

Supplementary Fig. S4. Optimization of CRISPR concentrations for engineering mutations. In the Table "CS" represents Cas9 and sgRNA concentration. As seen below, serial two fold deletions of CS starting with 100 ng/µl of Cas9 mRNA and 50 ng/µl sgRNA resulted in deletions in the *PRNP* locus, which was confirmed by cloning the PCR amplicons into PCR2.1 cloning vector and sequencing of 5 bacterial clones.

Cas9/sgRNA	CS 1	CS 2	CS3
Cas9	100	50	25
sgRNA (PRNP)	50	25	12.5

Crispr-Cas9&sgRNA(PRNP)-CS1

```
PRNP      CTGGGGCAGTGGTAGGGGGCCTCGGCGGTTACAT-GCTGGGGAGTGCCATGAGCAG
1-1       CTGGGGCAGTGGTAGGGGGCCTCGGCGGTTAC--TGCTGGGGAGTGCCATGAGCAG
1-2       CTGGGGCAGTGGTAGGGGGCCTCGGCGGTTAC--TGCTGGGGAGTGCCATGAGCAG
2-1       CTGGGGCAGTGGTAGGGGGCCTCGGCGGTTACATTGCTGGGGAGTGCCATGAGCAG
2-2       CTGGGGCAGTGGTAGGGGGCCTCGGCGGTTACATTGCTGGGGAGTGCCATGAGCAG
3-1       CTGGGGCAGTGGTAGGGGGCCTCGGC-----------TGGGGAGTGCCATGAGCAG
3-2       CTGGGGCAGTGGTAGGGGGCCTCGGC--------------GGAGTGCCATGAGCAG
4-1       CTGGGGCAGTGGTAGGGGGCCTCGGCGGTTACAT--CTGGGGAGTGCCATGAGCAG
4-2       CTGGGGCAGTGGTAGGGGGCCTCGGCGGTTACAT--CTGGGGAGTGCCATGAGCAG
5-1       CTGGGGCAGTG-----------------------------------CCATGAGCAG
5-2       CTGGGGCAGTG-----------------------------------CCATGAGCAG
6-1       CTGGGGCAGTGGTAGGGGGCCTCGGCC---TCATGGCTGGGGAGTGCCATGAGCAG
6-2       CTGGGGCAGTGGTAGGGGGCCTCGGCGGTTACATTGCTGGGGAGTGCCATGAGCAG
7-1       CTGGGGCAGTGGTAGGGGGCCTCGGCGGTTACATTGCTGGGGAGTGCCATGAGCAG
7-2       CTGGGGCAGTGGTAGGGGGCCTCGGCGGTTACATTGCTGGGGAGTGCCATGAGCAG
8-1       CTGGGGCAGTGGTAGGGGGCCTCGGCGGTTACATTGCTGGGGAGTGCCATGAGCAG
8-2       CTGGGGCAGTGGTAGGGG-CCTCGGCGGTTACATTGCTGGGGAGTGCCATGAGCAG
```

Crispr-Cas9&sgRNA(PRNP)-CS2

```
PRNP   GAAGCATGTGGCAGGCGCCGCTGCAGCTGGGGCAGTGGTAGGGGGCCTCGGCGGTTACATG-CTGGGGAG
1-1    GAAGCATGTGGCAGGCGCCGCTGCAGCTGGGGCAGTGGTAGGGGGCCTCGGCGGTTACATGGCCGGGGAG
1-2    GAAGCATGTGGCAGGCGCCGCTGCAGCTGGGGCAGTGGTAGGGGGCCTCGGCGGTTACATGGCTGGGGAG
2-1    GAAGCATGTGGCAGGTGCCGCTGCAGCTGGGGCAGTGGTAGGGGGCCTCGGCGGTTACA-G-CTGGGGAG
2-2    GAAGCATGTGGCAGGCGCCGCTGCAGCTGGGGCAGTGGTAGGGGGCCTCGGCGGTTACA-G-CTGGGGAG
3-1    GAAGCATGTGGCAGGCGCCGCTGCAGCTGGGGCAGTGGTAGGGGGCCTCGGCGGTTA-ATG-CTGGGGAG
3-2    GAAGCATGTGGCAGGCGCCGCTGCAGCTGGGGCAGTGGTAGGGGGCCTCGGCGGTTA-ATG-CTGGGGAG
4-1    GAAGCATGTGGCAGGCGCCGCTGCAGCTGGGGCAGTGGTAGGGGGCCTCGGCGGTTAC-TG-CTGGGGAG
4-2    GAAGCATGTGGCAGGCGCCGCTGCAGCTGGGGCAGTGGTAGGGGGCCTCGGCGGTTAC-TG-CTGGGGAG
5-1    GAGC-----------------------------------------------------------TGGGGAG
5-2    GAGC-----------------------------------------------------------TGGGGAG
6-1    GAAGC----------------------------------------------------------TGGGGAG
6-2    GAAGC----------------------------------------------------------TGGGGAG
7-1    GAAGCATGTGGCAGGCGCCGCTGCAGCTGGGGCAGTGGTAGGGGGCCTCGGCGGTTATATG-CTGGGGAG
7-2    GAAGCATGTGGCAGGCGCCGCTGCAGCTGGGGCAGTGGTAGGGGGCCTCGGCGGTTATATG-CTGGGGAG
8-1    GAAGCATGTGGCAGGCGCCGCTGCAGCTGGGGCAGTGGTGGGGGGCCTCGGCGGTTACATG-CTGGGGAG
8-2    GAAGCATGTGGCAGGCGCCGCTGCAGCTGGGGCAGTGGTAGGGGGCCTCGGCGGTTACATG-CTGGGGAG
9-1    GAAGCATGTGGCAGGCGCCGCTGCAGCTGGGGCAGTGGTAGGGGGCCTCGGCGGTTACA--GCTGGGGAG
9-2    GAAGCATGTGGCAGGCGCCGCTGCAGCTGGGGCAGTGGTAGGGGGCCTCGGCGGTTAC-------GGGAG
```

Crispr-Cas9&sgRNA(PRNP)-CS3

```
PRNP    CATGTGGCAGGCGCCGCTGCAGCTGGGGCAGTGGTAGGGGGCCTCGGCGGTTACAT-GCTGGGGAGTG
1-1     CATGAGGCAGGCGCCGCTGCAGCTGGGGCAGTGGTAGGGGGCCTCGGCG-----------GGGGAGTG
1-2     CATGTGGCAGGCGCCGCTGCAGCTGGGGCAGTGGTAGGGGGCCTCGGCGGTTACA--GCTGGGGAGTG
2-1     CATGTGGCAGGCGCCGCTGCAGCTGGGGCAGTGGTAGGGGGCCTCGGCGGTTACATTGCTGGGGAGTG
3-1     CATGTGGCAGGCGCCGCTG---------------------------G---------------GGAGTG
3-2     CATGT-----------------------------------------G---------------------
4-1     CATGTGGCAGGCGCCGCTGCAGCTGGGGCAGTGGTAGGGGGCCTCGGC------------GGGGAGTG
4-2     CATGTGGCAGGCGCCGCTGCAGCTGGGGCAGTGGCAGGGGGCCTCGGC------------GGGGAGTG
5-1     CATG--GCAGG------T--A-----------------------------------------------
6-1     CATGTGGCAGGCGCCGCTGCAGCTGGGGCAGTGGTAGGGGGCCTCGGC-----------TGGGGAGTG
7-1     CATGTGGCAGGCGCCGCTGCAGCTGGGGCAGTGGTAGGGGGCCTCGGCGGTTACATTGCTGGGGAGTG
8-1     CATG--GCAGG------T--A-----------------------------------------------
8-2     CATG--GCAGG------T--A-----------------------------------------------
10-1    CATGTGGCAGGCGCCGCTGCAGCTGGGGCAGTGGTAGGGGGCCTCGGC-----------TGGGGAGTG
10-2    CATGTGGCAGGCGCCGCTGCAGCTGGGGCAGTGGTAGGGGGCCTCGGC-----------TGGGGAGTG
11-1    CATGTGGCAGGCGCCGCTGCAGCTGGGGCAGTGGTAGGGGGCCTCGGCGGTTACA---CTGGGGAGTG
11-2    CATGTGGCAGGCGCCGCTGCAGCTGGGGCAGTGGTAGGGGGCCTCGGCGGTTACA---CTGGGGAGTG
```

Supplementary Fig. S5. A) Targeted knock-in of GFP expression vectors by injections into porcine embryos using Cas9 nickase. Top panel: Fluorescent micrograph showing GFP expressing embryos. Middle panel: Gel of PCR products showing the expected sized amplicon. The PCR amplicons (1050 bp) were cloned into PCR2.1 cloning vector and sequenced to confirm site-specific knock-in. Bottom panel: Schematic of *PRNP* and *GFP* sequence in the amplicons.

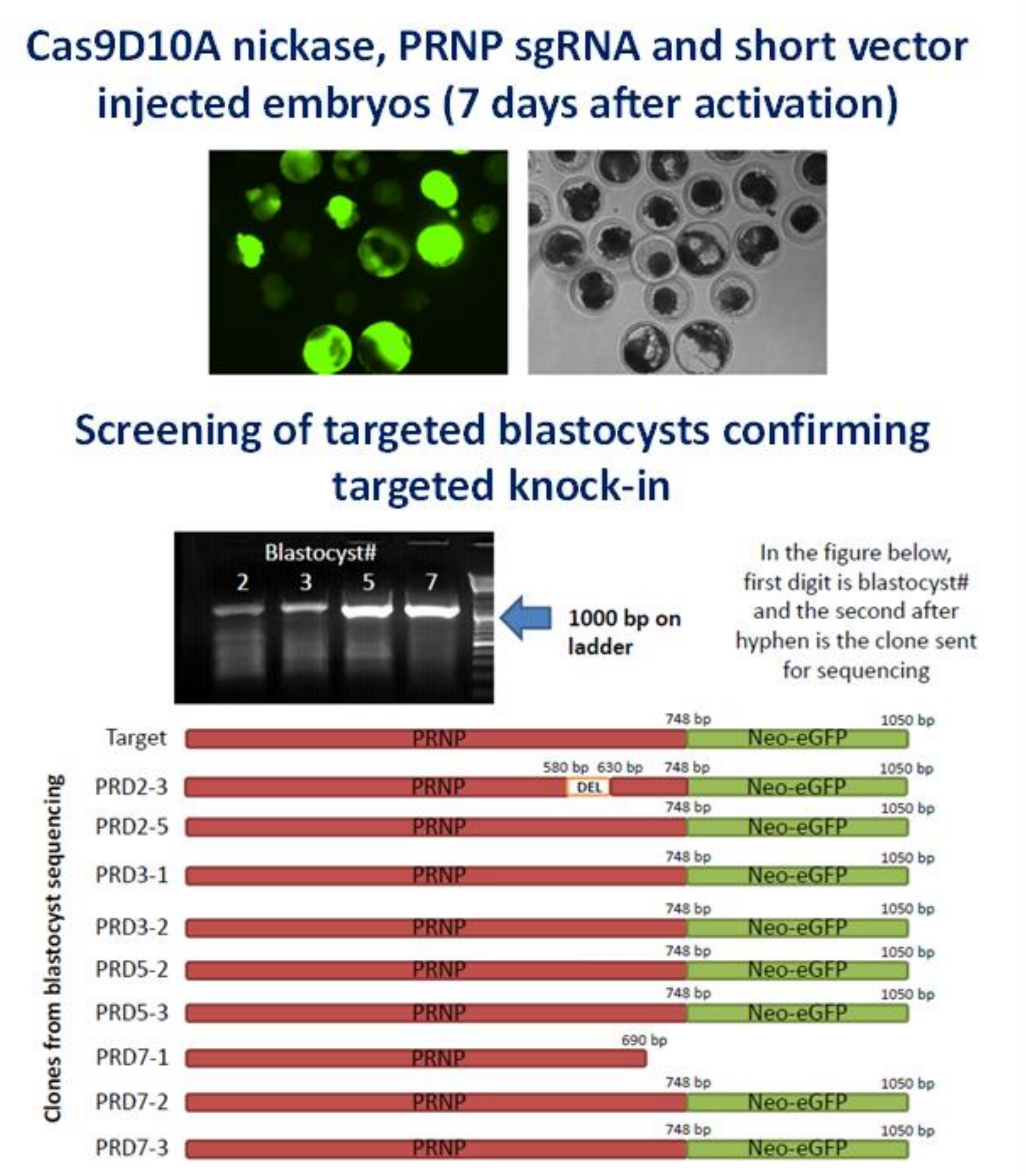

Supplementary Fig. 5B) Targeted knock-in of *GFP* expression vectors by injections into porcine embryos using Cas9 nuclease. Top panel: Fluorescent micrograph showing GFP expressing embryos. Middle panel: Gel of PCR products showing the expected sized amplicon. The PCR amplicons (1050 bp) and truncation products were cloned into PCR2.1 cloning vector and sequenced to confirm site-specific knock-in. Bottom panel: Schematic of *PRNP* and *GFP* sequence in the amplicons. Bottom panel: Schematic of *PRNP* and *GFP* sequence in the amplicons.

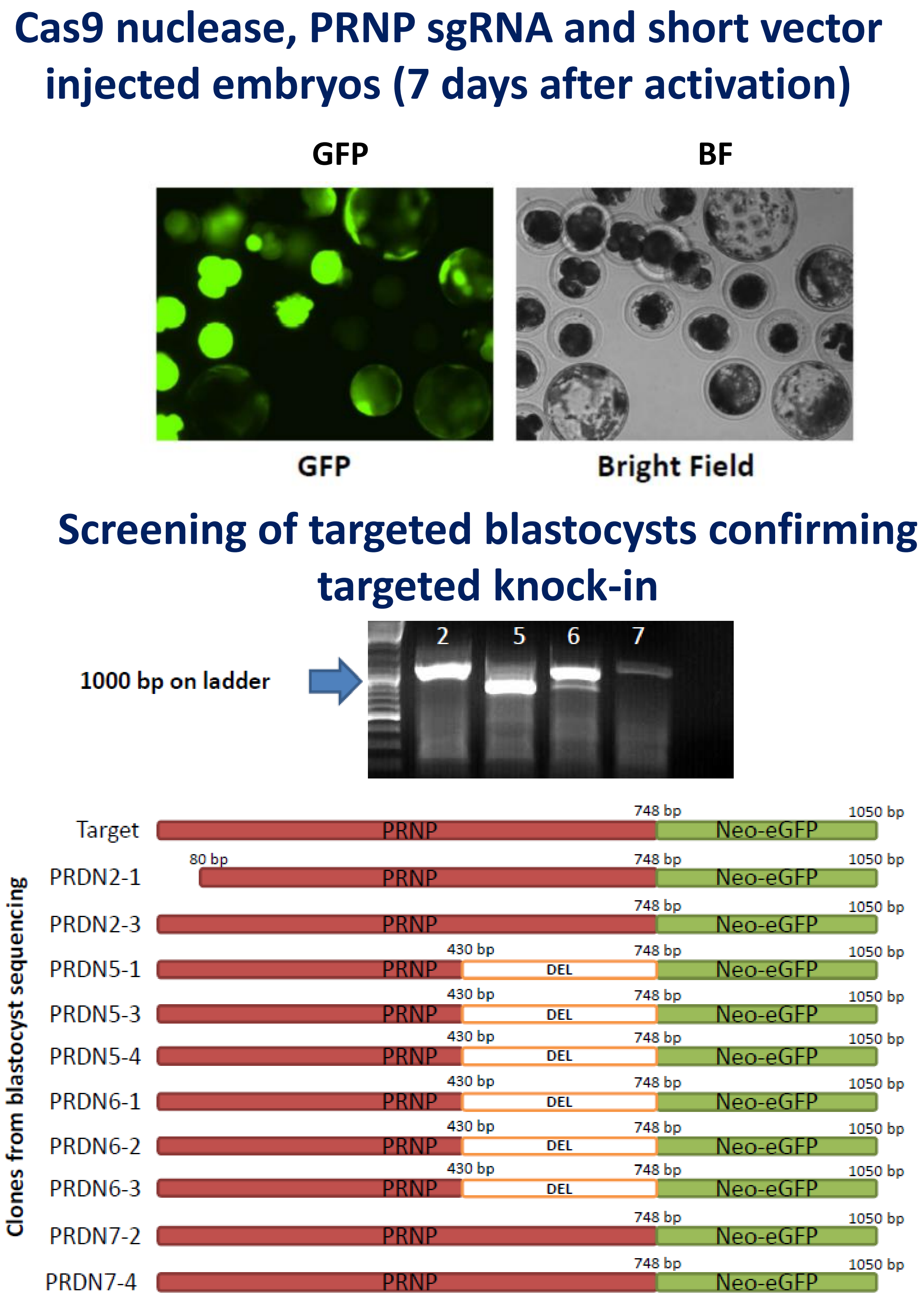

Supplementary Fig. 5C) The PCR amplicons were cloned into PCR2.1 cloning vector and sequenced to confirm site-specific knock-in by Sanger sequencing. The sequences were shown below.

PRD2-3

PRNP EGFP

Cas9-PRNP sgRNA-short vector/PRD2-3
>131211-18_M09_PRD2-3-T7promoter.ab1 1465
GGGGGGCCNNAANNNNGGCTCGAGCGGCCGCCAGTGTGATGGATATCTGC
AGAATTCGGCTNGCTCAAAGCTGTGGCAAACCCACCAGGTGTTTATTGTT
TTTTCCAGTTTAGATACAATGTATCAAGTAGAGGTTATTTTTACCATAAG
CATGTTGCTGGCATTCCACCTTTATCTTTTCTAAGAAACAGAGCCAGAAA
ATTATCTGAAGGTCAAATTTGTCCTTAGAGAAGGAGAAAGAGTTAACCCT
TCACCTACAGTTGTTTTTGTTGTAAGTGTTGCACAGGAGACAAATGGAGT
ATAAAGAACATTCACAGCTGATGCCACTACTATGTTCATTATGCTGCAGA
CATTAAGTGATTTCAATATAAACAGGACACTGACACCCTCTTTATTTTGT
ATTTTGCAGATAAGTAATCATGGTGAAAAGCCATATAGGTGGCTGGATCC
TCGTTCTCTTTGTGGCCGCATGGAGTGACATAGGGCTCTGCAAGAAGCGA
CCAAAGCCTGGCGGAGGATGGAACACTGGGGGGAGCCGATACCCAGGGCA
GGGTAGTCCTGGAGGCAACCGCTATCCACCCCAGGGAGGGGGTGGCTGGG
GACAGCCCCATGGAGGTGGCTGGGGACAGCCCCATGGTGGCGGAGGCTGG
GGTCAAGGTGGTGGCTCCCACGGTCAGTGGAACAAGCCCAGTAAGCCGAA
AACCAACATGAAGCATGTGGCAGGTGCCGCTGCAGCTGGGGCAGTGGTAG
GCGTACGTTAAAGATAATCATGCGTAAAATTGACGCATGTGTTTTATCGG
TCTGTATATCGAGGTTTATTTATTAATTTGAATAGATATTAAGTTTTATT
ATATTTACACTTACATACTAATAATAAATTCAACAAACAATTTATTTATG
TTTATTTATTTATTAAAAAAAAACAAAAACTCAAAATTTCTTCTATAAAG
TAACAAAACTTTTAACTAGTATGTCGAGGGACCTGATAACTTCGTATAGC
ATACATTATACGAAGTTATATTAAGGGTTCCGCAAGCTCTAGTCGAGCCC
CAGCTGGAAGCCGAATTCCAGCACACTGGCGGCCGTTACTAGTGGATCCG

PRD2-5(Reverse)

PRNP EGFP

Cas9-PRNP sgRNA-short vector/PRD2-5R
>131211-18_C09_PRD2-5-T7promoter.ab1 1518
NNNNNGGCCNNAANTGCATGCTCGAGCGGCCGCCAGTGTGATGGATATCT
GCAGAATTCCCAGCTGGGGCTCGACTAGAGCTTGTGGAACCCTTAATATA
ACTTCGTATAATGTATGCTATACGAAGTTATTAGGTCCCTCGACATACTA
GTTAAAAGTTTTGTTACTTTATAGAAGAAATTTTGGGTTTTTGTTTTTTT
TTAATAAATAAATAAACATAAATAAATTGTTTGTTGAATTTATTATTAGT
ATGTAAGTGTAAATATAATAAAACTTAATATCTATTCAAATTAATAAATA
AACCTCGATATACAGACCGATAAAACACATGCGTCAATTTTACGCATGAT
TATCTTTAACGTACGCCTACCACTGCCCCAGCTGCAGCGGCACCTGCCAC
ATGCTTAATGTTGGTTTTCGGCTCACTGGGCTTGTTCCACTGACCGTGGG
AGCCACCACCTTGACCCCAGCCTCCGCCACCATGGGGCTGTCCCCAGCCG
CCACCGTGGGGCTGTCCCCAGCCGCCTCCGTGGGGCTGTCCCCAGCCACC
TCCGCGGGGCTGTCCCCAGCCACCCCCTCCCTGGGGTGGATAGCGGTTGC
CTCCAGGACTACCCTGCCCTGGGTATCGGCTCCCCCCAGTGTTCCATCCT
CCGCCAGGCTTTGGTCGCTTCTTGCAGAGCCCTATGTCACTCCATGCGGC
CACAAAGAGAACGAGGATCCAGCCACCTATATGGCTTTTCACCATGATTA
CTTATCTGCAAAATACAAAATAAAGAGGGTGTCAGTGTCCTGTTTATATT
GAAATCACTTAATGTCTGCAGCATAATGAACATAGTAGTGGCATCAGCTG
TGAATGTTCTTTATACTCCATTTGTCTCCTGTGCAACACTTACAACAAAA
ACAACTGTAAGGTGAAGGGTTAACTCTTTCTCCTTCTCTAAGGACAAATT
TGACCTTCAGATAATTTTCTGGCTCTGTTTCTTAGAAAAGATAAAGGTGG
AATGCCAGCAGCATGCTTATGGTAAAAATAACCTACTTGATACATTGTAT
CTAAACTGGAAAAACAATAAACACCNTGGTGGGTTTGCCACAGCTTTGAG
CAAGCCGAATTCCAGCACACTGGCGGCCGTTACTAGTGGATCCGAGCTCG

PRD3-1

Cas9-PRNP sgRNA-short vector/PRD3-1

PRNP EGFP

>131205-09_E17_PRD3-1-T7promoter.ab1 1408
NNNNGGNNNNNNATGCTGCTCGAGCGGCCGCCAGTGTGATGGATATCTGCA
GAATTCGGCTTGCTCAAAGCTGTGGCAAACCCACCAGGTGTTTATTGTTT
TTTCCAGTTTAGATACAATGTATCAAGTAGAGGTTATTTTTACCATAAGC
ATGTTGCTGGCATTCCACCTTTATCTTTTCTAAGAAACAGAGCCAGAAAA
TTATCTGAAGGTCAAATTTGTCCTTAGAGAAGGAGAAAGAGTTAACCCTT
CACCTACAGTTGTTTTTGTTGTAAGTGTTGCACAGGAGACAAATGGAGTA
TAAAGAACATTCACAGCTGATGCCACTACTATGTTCATTATGCTGCAGAC
ATTAAGTGATTTCAATATAAACAGGACACTGACACCCTCTTTATTTTGTA
TTTTGCAGATAAGTAATCATGGTGAAAAGCCATATAGGTGGCTGGATCCT
CGTTCTCTTTGTGGCCGCATGGAGTGACATAGGGCTCTGCAAGAAGCGAC
CAAAGCCTGGCGGAGGATGGAACACTGGGGGGAGCCGATACCCAGGGCAG
GGTAGTCCTGAGGCAACCGCTATCCACCCCAGGGAGGGGGTGGCTGGGGC
CAGCCCCACGGAGGTGGCTGGGGACAGCCCCACGGAGGCGGCTGGGGACA
GCCCCACGGTGGCGGCTGGGGACAGCCCCATGGTGGCGGAGGCTGGGGTC
GAGGTGGTGGCTCCCACGGTCAGTGGAACAAGCCCAGTAAGCCGAAAACC
AACATGAAGCATGTGGCAGGTGCCGCTGCAGCTGGGGCAGTGGTAGGCGT
ACGTTAAAGATAATCATGCGTAAAATTGACGCATGTGTTTTATCGGTCTG
TATATCGAGGTTTATTTATTAATTTGAATAGATATTAAGTTTTATTATAT
TTACACTTACATACTAATAATAAATTCAACAAACAATTTATTTATGTTTA
TTTATTTATTAAAAAAAAACAAAAACTCAAAATTTCTTCTATAAAGTAAC
AAAACTTTTAACTAGTATGTCGAGGGACCTAATAACTTCGTATAGCATAC
ATTATACGAAGTTATTATTAAGGGTTCCGCAAGCTCTAGTCGAGCCCCAG
CTGGAAGCCGAATTCCAGCACACTGGCGGCCGTTACTAATGGATCCGAAC
TCGGTACCAAGCTTGGCGGAATCATGGGCNNNAGCTGTTTCCNGGGGGNA

PRD3-2

Cas9-PRNP sgRNA-short vector/PRD3-2
>131205-09_G17_PRD3-2-T7promoter.ab1

PRNP EGFP

GGNTGNNCCNNNAATGCATGCTCGAGCGGCCGCCAGTGTGATGGATATCT
GCAGAATTCGGCTTGCTCAAAGCTGTGGCAAACCCACCAGGTGTTTATTG
TTTTTTCCAGTTTAGATACAATGTATCAAGTAGAGGTTATTTTTACCATA
AGCATGTTGCTGGCATTCCACCTTTATCTTTTCTAAGAAACAGAGCCAGA
AAATTATCTGAAGGTCAAATTTGTCCTTAGAGAAGGAGAAAGAGTTAACC
CTTCACCTACAGTTGTTTTTGTTGTAAGTGTTGCACAGGAGACAAATGGA
GTATAAAGAACATTCACAGCTGATGCCACTACTATATTCATTATGCTGTA
GACATTAAGTGATTTCAATATAAACAGGACACTGACACCCTCTTTATTTT
GTATTTTGCAGATAAGTAATCATGGTGAAAAACCATATAGGTGGCTGGAT
CCTCGTTCTCTTTGTGGCCGCATGGAGTGACATAGGGCTCTGCAAGAAGC
GACCAAAGCCTGGCGGAGGATGGAACACTGGGGGGAGCCGATACCCAGGG
CAGGGTAGTCCTGGAGGCAACCGCTATCCACCCCAGGGAGGGGGTGGCTG
GGGACAGCCCCACGGAGGTGGCTGGGGACAGCCCCACGGAGGCGGCTGGG
GACAGCCCCACGGTGGCGGCTGGGGACAGCCCCATGGTGGCGGAGGCTGG
GGTCAAGGTGGTGGCTCCCACGGTCAGTGGAACAAGCCCAGTAAGCCGAA
AACCAACATGAAGCATGTGGCAGGTGCCGCTGCAGCTGGGGCAGTGGTAG
GCGTACGTTAAAGATAATCATGCGTAAAATTGACGCATGTGTTTTATCGG
TCTGTATATCGAGGTCTATTTATTAATTTGAATAGATATTAAGTTTTATT
ATATTTACACTTACATACTAATAATAAATTCAACAAACAATTTATTTATG
NTTAATTTATTTATTAAAAAAAAACAAAAACTCAAAATTTCTTCTATAAA
GTAACAAAACTTTTAACTAGTAATGTCGAGGGACCTAATAACTTCGTATA
GCATACATTATACGAAGTTATATTTAAAGGGTTCCGCAAGCTCTATTCGA
GCCCCACCTGGAAGCCGAATTCCAGCCACTGGCGGCGGTNNNTAATTGGA
TCCCAANTCCGGACCAAACTTGGCGGATCATGGGCAAAACTGTTCTCCNG

PRD5-2(Reverse)

PRNP EGFP

Cas9-PRNP sgRNA-short vector/PRD5-2R
>131205-09_A19_PRD5-2-T7promoter.ab1 1311
GGNTNGCNNTGANGATGCTCGAGCGGCCGCCAGTGTGATGGATATCTGCA
GAATTCGGCTTCCAGCTGGGGCTCGACTAGAGCTTGCGGAACCCTTAATA

TAACTTCGTATAATGTATGCTATACGAAGTTATTAGGTCCCTCGACATAC
TAGTTAAAAGTTTTGTTACTTTATAGAAGAAATTTTGAGTTTTTGTTTTT
TTTTAATAAATAAATAAACATAAATAAATTGTTTGTTGAATTTATTATTA
GTATGTAAGTGTAAATATAATAAAACTTAATATCTATTCAAATTAATAAA
TAAACCTCGATATACAGACCGATAAAACACATGCGTCAATTTTACGCATG
ATTATCTTTAACGTACGCCTACCACTGCCCCAGCTGCAGCGGCACCTGCC
ACATGCTTCATGTTGGTTTTCGGCTTACTGGGCTTGTTCCACTGACCGTG
GGAGCCACCACCTTGACCCCGGCCTCCGCCACCATGGGGCTGTCCCCAGC
CGCCACCGTGGGGCTGTCCCCAGCCGCCTCCGTGGGGCTGTCCCCAGCCA
CCTCCGTGGGGCTGTCCCCAGCCACCCCCTCCCTGGGGTGGATAGCGGTT
GCCTCCAGGACTACCCTGCCCTGGGTATCGGCTCCCCCCAGTGTTCCATC
CTCCGCCAGGCTTTGGTCGCTTCTTGCAGAGCCCTATGTCACTCCATGCG
GCCACAAAGAGAACGAGGATCCAGCCACCTATATGGCTTTTCACCATGAT
TACTTATCTGCAAAATACAAAATAAAGAGGGTGTCAGTGTCCTGTTTATA
TTGAAATCACTTAATGTCTGCAGCATAATGAACATAGTAGTGGCATCAGC
TGTGAATGTTCTTTATACTCCATTTGTCTCCTGTGCAACACTTACAACAA
AAACAACTGTAGGTGAAGGGTTAACTCTTTCTCCTTCTCTAAGGACAAAT
TTGACCTTCAGATAATTTTCTGGCTCTGTTTCTTAGAAAAGATAAAGGTG
GAATGCCAGCAACATGCTTATGGTAAAAATAACCTCTACTTGATACATTG
TATCTAAACTGGAAAANCAATAAACACCTGGTGGGTTTGCCACAGCTTTG
AACAAGCCGAATTCCAGCCACTGGCGGCCGTTACTAATGGATCCAANCTC
GGACCAAACTTGGCGAAACATGGGCAAAACCGGTTCTCGNNNGAAANNGT

PRD5-3(Reverse)

PRNP EGFP

Cas9-PRNP sgRNA-short vector/PRD5-3R
>131205-09_C19_PRD5-3-T7promoter.ab1 1353
GGNTGGCCNNNGATNCTGCTCGAGCGGCCGCCAGTGTGATGGATATCTGC
AGAATTCGGCTTCCAGCTGGGGCTCGACTAGAGCTTGCGGAACCCTTAAT
ATAACTTCGTATAATGTATGCTATACGAAGTTATTAGGTCCCTCGACATA
CTAGTTAAAAGTTTTGTTACTTTATAGAAGAGATTTTGAGTTTTTGTTTT
TTTTTAATAAATAAATAAACATAAATAAATTGCTTGTTGAGTTTATTATT
AGTATGTAAGTGTAAATATAATAAAACTTAATATCTATTCAAATTAATAA
ATAAACCTCGATATACAGACCGATAAAACACATGCGTCAATTTTACGCAT
GATTATCTTTAACGTACGCCTACCACTGCCCCAGCTGCAGCGGCACCTGC
CACATGCTTCATGTTGGTTTTCGGCTTACTGGGCTTGTTCCACTGACCGT
GGGAGCCACCACCTTGACCCCAGCCTCCGCCACCATGGGGCTGTCCCCAG
CCGCCACCGTGGGGCTGTCCCCAGCCGCCTCCGTGGGGCTGTCCCCAGCC
ACCTCCGTGGGGCTGTCCCCAGCCACCCCCTCCCTGGGGTGGATAGCGGT
TGCCTCCAGGACTACCCTGCCCTGGGTATCGGCTCCCCCCAGTGTTCCAT
CCTCCGCCAGGCTTTGGTCGCTTCTTGCAGAGCCCTATGTCACTCCATGC
GGCCACAAAGAGAACGAGGATCCAGCCACCTATGTGGCTTTTCACCATGA
TTACTTATCTGCAAAATACAAAATAAAGAGGGTGTCAGTGTCCTGTTTAT
ATTGAAATCACTTAATGTCTGCAGCATATTGAACATAGTAGTGGCATCAG
CTGTGAATGTTCTTTATACTCCATTTGTCTCCTGTGCAACACTTACAACA
AAAACAACTGTAGGTGAAGGGTTAACTCTTTCTCCTTCTCTAAGGACAAA
TTTGACCTTCAGATAATTTTCTGGCTCTGTTTCTTAGAAAAGATAAAGGT
GGAATGCCAGCAACATGCTTATGGTAAAAATAACCTCTACTTGATACATT
GTATCTAAACTGGAAAAACAATAAACACCTGGGNGNTTNGCCACAGCTTT
GAGCAAGCCGAATTCCAGCCACTGGCGGCCGTTACTAATGGATCCGAACC
GGGACCAANCTTGGNGAAACATGGGCAAANCGGTTNCTCGGGTGGAAATT

PRD7-1

PRNP

Cas9-PRNP sgRNA-short vector/PRD7-1
>131211-18_K07_PRD7-1-T7promoter.ab1 1514
NNNNNNNNNNANATGCATGCTCGAGCGGCCGCCAGTGTGATGGATATCTG
CAGAATTCGGCTTGCTCAAAGCTGTGGCAAACCCACCAGGTGTTTATTGT
TTTTTCCAGTTTAGATACAATGTATCAAGTAGAGGTTATTTTTACCATAA
GCATGTTGCTGCATTCCACCTTTATCTTTTCTAAGAAACAGAGCCAGAAA
ATTATCTGAAGGTCAAATTTGTCCTTAGAGAAGGAGAAAGAGTTAACCCT
TCACCTACAGTTGTTTTTGTTGTAAGTGTTGCACAGGAGACAAATGGAGT

ATAAAGAGCATTCACAGCTGATGCCACTACTATGTTCATTATGCTGCAGA
CATTAAGTGATTTCAATATAAACAGGACACTGACACCCTCTTTATTTTGT
ATTTTGCAGATAAGTAATCATGGTGAAAAGCCATATAGGTGGCTGGATCC
TCGTTCTCTTTGTGGCCGCATGGAGTGACATAGGGCTCTGCAAGAAGCGA
CCAAAGCCTGGCGGAGGATGGAACACTGGGGGGAGCCGATACCCAGGGCA
GGGTAGTCCTGGAGGCAACCGCTTTCCACCCCAGGGAGGGGGTGGCTGGG
GACAGCCCCACGGAGGTGGCTGGGGACAGCCCCACGGAGGCGGCTGGGGA
CAGCCCCACGGTGGCGGCTGGGGACAGCCCCATGGTGGCGGAGGCTGGGG
TCAAGGTGGTGGCTCCCACGGTCAGTGGAACAAGCCGAATTCCAGCACAC
TGGCGGCCGTTACTAGTGGATCCGAGCTCGGTACCAAGCTTGGCGTAATC

PRD7-2

PRNP EGFP

Cas9-PRNP sgRNA-short vector/PRD7-2
>131211-18_O09_PRD7-2-T7promoter.ab1 1413
NNNNNGGCCNNANNTGCTGCTCGAGCGGCCGCCAGTGTGATGGATATCTG
CAGAATTCGGCTTGCTCAAAGCTGTGGCAAACCCACCAGGTGTTTATTGT
TTTTTCCAGTTTAGATACAATGTATCAAGTAGAGGTTATTTTTACCATAA
GCATGTTGCTGGCATTCCACCTTTATCTTTTCTAAGAAACAGAGCCAGAT
AATTATCTGAAGGTCAAATTTGTCCTTAGAGAAGGAGAAAGAGTTAACCC
TTCACCTACAGTTGTTTTTGTTGTAAGTGTTGCACAGGAGACAAATGGAG
TATAAAGAACATTCACAGCTGATGCCACTACTATGTTCATTATGCTGCAG
ACATTAAGTGATTTCAATATAAACAGGACACTGACACCCTCTTTATTTTG
TATTTTGCAGATAAGTAATCATGGTGAAAAGCCATATAGGTGGCTGGATC
CTCGTTCTCTTTGTGGCCGCATGGAGTGACATAGGGCTCTGCAAGAAGCG
ACCAAAGCCTGGCGGAGGATGGAACACTGGGGGGAGCCGATACCCAGGGC
AGGGTAGTCCTGGAGGCAACCGCTATCCACCCCAGGGAGGGGGTGGCTGG
GGACAGCCCCACGGAGGTGGCTGGGGACAGCCCCACGGAGGCGGCTGGGG
ACAGCCCCACGGTGGCGGCTGGGGACAGCCCCATGGTGGCGGAGGCTGGG
GTCAAGGTGGTGGCTCCCACGGTCAGTGGAACAAGCCCAGTAAGCCGAAA
ACCAACATGAAGCATGTGGCAGGTGCCGCTGCAGCTGGGGCAGTGGTAGG
CGTACGTTAAAGATAATCATGCGTAAAATTGACGCATGTGTTCTATCGGT
CTGTATATCGAGGTTTATTTATTAATTTGAATAGATATTAAGTTTTATTA
TATTTACACTTACATACTAATAATAAATTCAACAAACAATTTATTTATGT
TTATTTATTTATTAAAAAAAACAAAAACTCAAAATTTCTTCTATAAAGTA
ACAAAACTTTTGACTAGTATGTCGAGGGACCTAATAACTTCGTATAGCAT
ACATTATACGAAGTTATATTAAGGGTTCCGCAAGCTCTAGTCGAGCCCCA
GCTGGAAGCCGAATTCCAGCACACTGGCGGCCGTTACTAGTGGATCCGAG
CTCGGTACCAAGCTTGGCGTAATCATGGGCATAGCTGTTTCCTGGNTGAA

PRD7-3 (Reverse)

PRNP EGFP

Cas9-PRNP sgRNA-short vector/PRD7-3R
>131211-18_M11_PRD7-3-T7promoter.ab1 1436
GGGGTGNCCNNTGATGNNGCTCGAGCGGCCGCCAGTGTGATGGATATCTG
CAGAATTCGGCTTCCAGCTGGGGCTCGACTAGAGCTTGCGGAACCCTTAA
TTTAACTTCGTATAATGTATGCTATACGAAGTTATTAGGTCCCTCGACAT
ACTAGTTAAAAGTTTTGTTACTTTATAGAAGAAATTTTGAGTTTTTGTTT
TTTTTTAATAAATAAATAAACATAAATAAATTGTTTGTTGAATTTATTAT
TAGTATGTAAGTGTAAATATAATAAAACTTAATATCTATTCAAATTAATA
AATAAACCTCGATATACAGACCGATAAAACACATGCGTCAATTTTACGCA
TGATTATCTTTAACGTACGCCTACCACTGCCCCAGCTGCAGCGGCACCTG
CCACATGCTTCATGTTGGTTTTCGGCTTACTGGGCTTGTTCCACTGACCG
TGGGGGCCACCACCTTGACCCCAGCCTCCGCCACCATGGGGCTGTCCCCA
GCCGCCACGNTGGGGCTGTCCCCAGCCGCCTCCGTGGGGCTGTCCCCAGC
CACCTCCGTGGGGCTGTCCCCAGCCACCCCCTCCCTGGGGTGGATAGCGG
TTGCCTCCAGGACTACCCTGCCCTGGGAATCGGCTCCCCCCAGTGTTCCA
TCCTCCGCCAGGCTTTGGTCGCTTCTTGCAGAGCCCTATGTCGCTCCATG
CGGCCACAAAGAGAACGAGGATCCAGCCACCTATATGGCTTTTCACCATG
ATTACTTATCTGCAAAATACAAAATAAAGAGGGTGTCAGTGTCCTGTTTA
TATTGAAATCACTTAATGTCTGCAGCATAATGAACATAGTAGTGGCATCA
GCTGTGAATGTTCTTTATACTCCATTTGTCTCCTGTGCAACACTTACAAC

AAAAACAACTGTAAGGTGAAGGGTTAACTCTTTCTCCTTCTCTAAGGACA
AATTTGACCTTCAGATAATTTTCTGGCTCTGTTTCTTAGAAAAGATAAAG
GTGGAATGCCAGCAACATGCTTATGGTAAAAATAACCTCTACTTGATACA
TTGTATCTAAACTGGAAAAACAATAAACACCTGGTGGGTTTGCACAGCTT
TGAGCAAGCCGAATTCCGCACACTGGCGGCCGTTACTAGTGGATCCGNAC
TCGGTACCAAGCTTGGNNNAATCATGGGCATAGCTGTTTCCTGGGGNAAA

PRDN2-1 (Reverse)

PRNP EGFP

Cas9-PRNP sgRNA-short vector/PRDN2-1R
>131211-18_C13_PRDN2-1-T7promoter.ab1 1568
GGTGGGCCNNTGATGCATGCTCGAGCGGCCGCCAGTGTGATGGATATCTG
CAGAATTCGGCTTCCAGCTGGGGCTCGACTAGAGCTTGCGGAACCCTTAA
TATAACTTCGTATAATGTATGCTATACGAAGTTATTAGGTCCCTCGACAT
ACTAGTTAAAAGTTTTGTTACTTTATAGAAGAAATTTTGAGTTTTTGTTT
TTTTTTAATAAATAAATAAACATAAATAAATTGTTTGTTGAATTTATTAT
TAGTATGTAAGTGTTAATATAATAAAACTTAATATCTATTCAAATTAATA
AATAAACCTCGATATACAGACCGATAAAACACATGCGTCAATTTTACGCA
TGATTATCTTTAACGTACGCCTACCACTGCCCCAGCTGCAGCGGCACCTG
CCACATGCTTCATGTTGGTTTTCGGCTTACTGGGCTTGTTCCACTGACCG
TGGGAGCCACCACCTTGACCCCAGCCTCCGCCACCATGGGGCTGTCCCCA
GCCGCCACCGTGGGGCTGTCCCCAGCCGCCTCCGTGGGGCTGTCCCCAGC
CATCTCCGTGGGGCTGTCCCCAGCCACCCCCTCCCTGGGGTGGACAGCGG
TTGCCTCCAGGACTACCCTGCCCTGGGCATCGGCTCCCCCCAGTGTTCCA
TCCTCCGCCAGGCTTTGGTCGCTTCTTGCAGAGCCCTATGTCACTCCATG
CGGCCACAAAGAGAACGAGGATCCAGCCACCTATATGGCTTTTCACCATG
ATTACTTATCTGCAAAATACAAAATAAAGAGGGTGTCAATGTCCTGTTTA
TATTGAAATCACTTAATGTCTGCAGCATAATGAACATAGTAGTGGCATCA
GCTGTGAATGTTCTTTATACTCCATTTGTCTCCTGTGCAACACTTACAAC
AAAAACAACTGGAAGGGTGAAGGGTTCGCGCCTTGATGCAATAGCTGCTG
TGTAACCTGTACTGCAAATTGGAAAAACAATAAACACATTGGTGGGTTTG
CCACAGCTTTGAGCAAGCCGAATTCCAGCACACTGGCGGCCGTTACTAGT
GGATCCGAGCTCGGTACCAAGCTTGGCGTAATCATGGGTCATAGCTGTTN

PRDN2-3

PRNP EGFP

Cas9-PRNP sgRNA-short vector/PRDN2-3
>131211-18_K13_PRDN2-3-T7promoter.ab1 1493
GAGANGNCNNTNNTGCTGCTCGAGCGGCCGCCAGTGTGATGGATATCTGC
AGAATTCGGCTTGTGGCAAACCCACCAGGTGTTTATTGTTTTTCCAGTT
TAGATACAATGTATCAAGTAGAGGTTATTTTTACCATAAGCATGTTGCTG
GCGTTCCACCTTTATCTTTTCTAAGAAACAGAGCCAGAAAATTATCTGAA
GGTCAAATTTGTCCTTAGAGAAGGAGAAAGAGTAAACCCTTCACCTACAG
TTGTTTTTGTTGTAAGTGTTGCACAGGAGACAAATGAAGTATAAAGAACA
TTCACAGCTGATGCCACTACTATGTTCATTATGCTGCAGACATTAAGTGA
TTTCAATATAAACAGGACACTGACACCCTCTTTATTTTGTATTTTGCAGA
TAAGTAATCATGGTGAAAAGCCATATAGGTGGCTGGATCCTCGTTCTCTT
TGTGGCCGCATGGAGTGACATAGGGCTCTGCAAGAAGCGACCAAAGCCTG
GCGGAGGATGGAACACTGGGGGGAGCCGATACCCAGGGCAGGGTAGTCCT
GGAGGCAACCGCTATCCACCCCAGGGAGGGGGGTGGGCTGGGGACAGCCC
CACGGAGGTGGCTGGGGACAGCCCCACGGAGGCGGCTGGGGACAGCCCCA
CGGTGGCGGCTGGGGACAGCCCCATGGTGGCGGAGGCTGGGGTCAAGGTG
GTGGCTCCCACGGTCAGTGGAACAAGCCCAGTAAGCCGAAAACCAACATG
AGGCATGCGGCAGGTGCCTCTGCAGCTGGGGCAGTGGTAGGCGTACGTTA
AAGATAATCATGCGTAAAATTGACGCATGTGTTTTATCGGTCTGTATATC
GAGGTTTATTTATTAATTTGAATAGATATTAAGTTTTATTATATTTACAC
TTACATACTAACAATAAATTCAACAAACAATTTATTTATGTTTATTTATT
TATTAAAAAAAAACAAAAACTCAAAATTTCTTCTATAAAGTAATAAAACT
TTTAACTAGTATGTCGAGGGACCTAATAACTTCGTATAGCATACATTATA
CGAAGTTATATTAAGGGTTCCGCAAGCTCTAGTCGAGCCCCAGCTGGAAG

CCGAATTCCAGCACACTGGCGGCCGTTACTAGTGGATCCGAGCTCGGTAC

PRDN5-1

PRNP EGFP

Cas9-PRNP sgRNA-short vector/PRDN5-1
>131205-09_E19_PRDN5-1-T7promoter.ab1 1409
NNGNNNGCTCTAATGCTGCTCGAGCGGCCGCCAGTGTGATGGATATCTGC
AGAATTCGGCTTGCTCAAAGCTGTGGCAAACCCACCAGGTGTTTATTGTT
TTTTCCAGTTTAGATACAATGTATCAAGTAGAGGTTATTTTTACCATAAG
CATGTTGCTGGCATTCCACCTTTATCTTTTCTAAGAAACAGAGCCAGAAA
ATTATCTGAAGGTCAAATTTGTCCTTAGAGAAGGAGAAAGAGTTAACCCT
TCACCTACAGTTGTTTTTGTTGTAAGTGTTGCACAGGAGACAGATGGAGT
ATAAAGAACATTCACAGCTGATGCCACTACTATGTTCATTATGCTGCAGA
CATTAAGTGATTTCAATATAAACGGGACACTGACACCCTCTTTATTTTGT
ATTTTGCAGATAAGTAATCATGGTGAAAAGCCATATAGGTGGCTGGATCC
TCGTTCTCTTTGTGGCCGCATGGAGTGGCGGCCGCATACGCGTATACTAG
ATTAACCCTAGAAAGATAATCATATTGTGACGTACGTTAAAGATAATCAT
GCGTAAAATTGACGCATGTGTTTTATCGGTCTGTATATCGAGGTTTATTT
ATTAATTTGAATAGATATTAAGTTCTATTATATTTACACTTACATACTAA
TAATAAATCCAACAAACAATTAATTTATGTTTATTTATTTATTAAAAAAA
AACAAAAACTCAAAATTTCTTCTATAAAGTAACAAAACTTTTAACTAGTA
TGTTGAGGGACCTAATAACTTCGTATAGCATACATTATACGAAGTTATAT
TAAGGGTTCCGCAAGCTCTAGTCGAGCCCCAGCTGGAAGCCGAATTCCAG
CACACTGGCGGCCGTTACTAGTGGATCCGAGCTCGGTACCAAGCTTGGCG

PRDN5-3 (Reverse)

PRNP EGFP

Cas9-PRNP sgRNA-short vector/PRDN5-3R
>131205-09_G19_PRDN5-3-T7promoter.ab1 1404
GGTGGCCNNTNANGATGCTCGAGCGGCCGCCAGTGTGATGGATATCTGCA
GAATTCGGCTTCCAGCTGGGGCTCGACTAGAGCTTGCGGAACCCTTAATA
TAACTTCGTATAATGTATGCTATACGAAGTTATTAGGTCCCTCAACATAC
TAGTTAAAAGTTTTGTTACTTTATAGAAGAAATTTTGAGTTTTTGTTTTT
TTTTAATAAATAAATAAACATAAATAAATTGTTTGTTGAATTTATTATTA
GTATGTAAGTGTAAATATAATAAAACTTAATATCTATTCAAATTGATAAA
TAAACCTCGATATACAGACCGATAAAACACATGCGTCAATGTTACGCATG
ATTATCTTTAACGTACGTCACAATATGATTATCTTTCTAGGGTTAATCTA
GTATACGCGTATGCGGCCGCCACTCCATGCGGCCACAAAGAGAACGAGGA
TCCAGCCACCTATATGGCTTTTCACCATGATTACTTATCTGCAAAATACA
AAATAAAGAGGGTGTCAGTGTCCTGTTTATATTGAAATCACTTAATGTCT
GCAGCATAATGAACATAGTAGTGGCATCAGCTGTGAATGTTCTTTATACT
CCATTTGTCTCCTGTGCAACACTTACAACAAAAACAACTGTAGGTGAAGG
GTTAACTCTTTCTCCTTCTCTAAGGACAAATTTGACCTTCAGATAATTTT
CTGGCTCTGTTTCTTAGAAAAGATAAAGGTGGAATGCCAGCAACATGCTT
ATGGTAAAAATAACCTCTACTTGATACATTGTATCTAAACTGGAAAAAAC
AATAAACACCTNGGTGGGTTTGCCACAGCTTTGAGCAAGCCGAATTCCAG
CACACTGGCGGCCGTTACTAGTGGATCCGAGCTCGGTACCAAGCTTGGCG

PRDN5-4

PRNP EGFP

Cas9-PRNP sgRNA-short vector/PRDN5-4
>131205-09_I19_PRDN5-4-T7promoter.ab1 1450
GCGTGNNNNNNAATGCATGCTCGAGCGGCCGCCAGTGTGATGGATATCTG
CAGAATTCGGCTTGCTCAAAGCTGTGGCAAACCCACCAGGTGTTTATTGT
TTTTTCCAGTTTAGATACAATGTATCAAGTAGAGGTTATTTTTACCATAA
GCATGTTGCTGCATTCCACCTTTATCTTTTCTAAGAAACAGAGCCAGAAA
ATTATCTGAAGGTCAAATTTGTCCTTAGAGAAGGAGAAAGAGTTAACCCT
TCACCTACAGTTGTTTTTGTTGTAAGTGTCGCACAGGAGACAAATGGAGT
ATAAAGAACATTCACAGCTGATGCCACTACTATGTTCATTATGCTGCAGA
CATTAAGTGATTTCAATATAAACAGGACACTGACACCCTCTTTATTTTGT

ATTTTGCAGATAAGTAATCATGGTGAAAAGCCATATAGGTGGCTGGATCC
TCATTCTCTTTGTGGCCGCATGGAGTGGCGGCCGCATACGCGTATACTAG
ATTAACCCTAGAAAGATAATCATATTGTGACGTACGTTAAAGATAATCAT
GCGTAACATTGACGCATGTGTTTTATCGGTCTGTATATCGAGGTTTATTT
ATTAATTTGAATAGATATTAAGTTTTATTATATTTACACTTACATACTAA
TAATAAATTCAACAAACAATTTATTTATGTTTATTTATTTATTAAAAAAA
AACAAAAACTCAAAATTTCTTCTATAAAGTAACAAAGCTTTTAACTAGTA
TGTTGAGGGACCTAATAACTTCGTATAGCATACATTATACGAAGTTATAT
TAAGGGTTCCGCAAGCTCTAGTCGAGCCCCAGCTGGAAGCCGAATTCCAG
CACACTGGCGGCCGTTACTAGTGGATCCGAGCTCGGTACCAAGCTTGGCG

PRDN6-1

PRNP EGFP

Cas9-PRNP sgRNA-short vector/PRDN6-1
>131211-18_K09_PRDN6-1-T7promoter.ab1 1478
NNNNCGGCCNNNNATGATGCTCGAGCGGCCGCCAGTGTGATGGATATCTG
CAGAATTCGGCTTGCTCAAAGCTGTGGCAAACCCACCAGGTGTTTATTGT
TTTTTCCAGTTTAGATACAATGTATCAAGTAGAGGTTATTTTTACCATAA
GCATGTTGCTGGCATTCCACCTTTATCTTTTCTAAGAAACAGAGCCAGAA
AATTATCTGAAGGTCAAATTTGTCCTTAGAGAAGGAGAAAGAGTTAACCC
TTCACCTACAGTTGTTTTTGTTGTAAGTGTTGCACAGGAGACAAATGGAG
TATAAAGAACATTCACAGCTGATGCCACTACTATGTTCATTATGCTGCAG
ACATTAAGTGATTTCAATATAAACAGGACACTGACACCCTCTTTATTTTG
TATTTTGCAGATAAGTAATCATGGTGAAAAGCCATATAGGTGGCTGGATC
CTCGTTCTCTTTGTGGCCGCATGGAGTGGCGGCCGCATACGCGTATACTA
GATTAACCCTAGAAAGATAATCATATTGTGACGTACGTTAAAGATAATCA
TGCGTAAAATTGACGCATGTGTTTTATCGGTCTGTATATCGAGGTTTATT
TATTAATTTGAATAGATATTAAGTTTTATTATATTTACACTTACATACTA
ATAATAAATTCAACAAACAATTTATTTATGTTTATTTATTTATTAAAAAA
AAACAAAAACTCAAAATTTCTTCTATAAAGTAACAAAACTTTTAACTAGT
ATGTCGAGGGACCTAATAACTTCGTATAGCATACATTATACGAAGTTATA
TTAAGGGTTCCGCAAGCTCTAGTCGAGCCCCAGCTGGGAATTCCAGCACA
CTGGCGGCCGTTACTAGTGGATCCGAGCTCGGTACCAAGCTTGGCGTAAT

PRDN6-2

PRNP EGFP

Cas9-PRNP sgRNA-short vector/PRDN6-2
>131211-18_K05_PRDN6-2-T7promoter.ab1 1467
NNNTGGCCNNNAATGCTGCTCGAGCGGCCGCCAGTGTGATGGATATCTGC
AGAATTCGGCTTGCTCAAAGCTGTGGCAAACCCACCAGGTGTTTATTGTT
TTTTCCAGTTTAGATACAATGTATCAAGTAGAGGTTATTTTTACCATAAG
CATGTTGCTGGCATTCCACCTTTATCCTTTCTAAGAAACAGAGCCAGAAA
ATTATCTGAAGGTCAAATTTGTCCTTAGAGAAGGAGAAAGAGTTAACCCT
TCACCTACAGTTGTTTTTGTTGTAAGTGTTGCACAGGAGACAAATGGAGT
ATAAAGAACATTCACAGCTGATGCCACTACTATGTTCATTATGCTGCAGA
CATTAAGTGATTTCAATATAAACAGGACACTGACACCCTCTTTATTTTGT
ATTTTGCAGATAAGTAATCATGGTGAAAAGCCATATAGGTGGCTGGATCC
TCGTTCTCTTTGTGGCCGCATGGAGTGGCGGCCGCATACGCGTATACTAG
ATTAACCCTAGAAAGATAATCATATTGTGACGTACGTTAAAGATAATCAT
GCGTAAAATTGACGCATGTGTTTTATCGGTCTGTATATCGAGGTTTATTT
ATTAACTTGAATAGATATTAAGTTTTATTATATTTACACTTACATACTAA
TAATAAATTCAACAAACAATTTATTTATGTTTATTTATTTATTAAAAAAA
AACAAAAACTCAAAATTTCTTCTATAAAGTAACAAAACTTTTAACTAGTA
TGTCGAGGGACCTAATAACTTCGTATAGCATACATTATACGAAGTTATAT
TAAGGGTTCCGCAAGCTCTAGTCGAGCCCCAGCTGGAAGCCGAATTCCAG
CACACTGGCGGCCGTTACTAGTGGATCCGAGCTCGGTACCAAGCTTGGCG

PRDN6-3

PRNP EGFP

Cas9-PRNP sgRNA-short vector/PRDN6-3
>131211-18_E11_PRDN6-3-T7promoter.ab1 1558
NNNNNGCCNNNAATGCTGCTCGAGCGGCCGCCAGTGTGATGGATATCTGC
AGAATTCGGCTTGCTCAAAGCTGTGGCAAACCCACCAGGTGTTTATTGTT
TTTTCCAGTTTAGATACAATGTATCAAGTAGAGGTTATTTTTACCATAAG
CATGTTGCTGGCATTCCACCTTTATCTTTTCTAAGAAACAGAGCCAGAAA
ATTATCTGAAGGTCAAATTTGTCCTTAGAGAAGGAGAAAGAGTTAACCCT
TCACCTACAGTTGTTTTTGCTGTAAGTGTTGCACAGGAGACAAATGGAGT
ATAAAGAACATTCACAGCTGATGCCACTACTATGTTCATTATGCTGCAGA
CATTAAGTGATTTCAATATAAACAGGACACTGACACCCTCTTTATTTTGT
ATTTTGCAGATAAGTAATCATGGTGAAAAGCCATATAGGTGGCTGGATCC
TCGTTCTCTTTGTGGCCGCATGGAGTGGCGGCCGCATACGCGTATACTAG
ATTAACCCTAGAAAGATAATCATATTGTGACGTACGTTAAAGATAATCAT
GCGTAAAATTGACGCATGTGTTTTATCGGTCTGTATATCGAGGTTTATTT
ATTAATTTGAATAGATATTAAGTTTTATTATATTTACACTTACATACTAA
TAATAAATTCAACAAACAATTTATTTATGTTTATTTATTTATTAAAAAAA
AACAAAAACTCAAAATTTCTTCTATAAAGTAACAAAACTTTTAACTAGTA
TGTCGAGGGACCTAATAACTTCGTATAGCATACATTATACGAAGTTATAT
TAAGGGTTCCGCAAGCTCTAGTCGAGCCCCAGCTGGAAGCCGAATTCCAG
CACACTGGCGGCCGTTACTAGTGGATCCGAGCTCGGTACCAAGCTTGGCG

PRDN7-2

PRNP EGFP

Cas9-PRNP sgRNA-short vector/PRDN7-2
>131211-18_I13_PRDN7-2-T7promoter.ab1 1556
NNNNGGCCNNTNATGCATGCTCGAGCGGCCGCCAGTGTGATGGATATCTG
CAGAATTCGGCTTGCTCAAAGCTGTGGCAAACCCACCAGGTGTTTATTGT
TTTTTCCAGTTTAGATACAATGTATCAAGTAGAGGTTATTTTTACCATAA
GCATGTTGCTGGCATTCCACCTTTATCTTTTCTAAGAAACAGAGCCAGAA
AATTATCTGAAGGTCAAATTTGTCCTTAGAGAAGGAGAAAGAGTTAACCC
TTCACCTACAGTTGTTTTTGTTGTAAGTGTTGCACAGGAGACAAATGGAG
TATAAAGAACATTCACAGCTGATGCCACTACTATGTTCATTATGCTGCAG
ACATTAAGTGATTTCAATATAAACAGGACACTGACACCCTCTTTATTTTG
TATTTTGCAGATAAGTAATCATGGTGAAAAGCCATATAGGTGGCTGGATC
CTCGTTCTCTTTGTGGCCGCATGGAGTGACATAGGGCTCTGCAAGAAGCG
ACCAAAGCCTGGCGGAGGATGGAACACTGGGGGGAGCCGATACCCAGGGC
AGGGTAGTCCTGGAGGCAACCGCTATCCACCCCAGGGAGGGGGTGGCTGG
GGACAGCCCCACGGAGGTGGCTGGGGACAGCCCCACGGAGGCGGCTGGGG
ACAGCCCCACGGTGGCGGCTGGGGACAGCCCCATGGTGGCGGAGGCTGGG
GTCAAGGTGGTGGCTCCCACGGTCAGTGGAACAAGCCCAGTAAGCCGAAA
ACCAACATGAAGCATGTGGCAGGTGCCGCTGCAGCTGGGGCAGTGGTAGG
CGTACGTTAAAGATAATCATGCGTAAAATTGACGCATGTGTTTTATCGGT
CTGTATATCGAGGTTTATTTATTAATTTGAATAGATATTAAGTTTTATTA
TATTTACACTTACATACTAATAATAAATTCAACAAACAATTTATTTATGT
TTATTTATTTATTAAAAAAAAACAAAAACTCAAAATTTCTTCTATAAAGT
AACAAAACTTTTAACGAGTATGTCGAGGGACCTAATAACTTCGTATAGCA
TACATTATACGAAGTTNATATTAAGGGTTCCGCAAGCTCTAGTCGAGCCC
CAGCTGGAAGCCGAATTCCAGCACACTGGCGGCNNGTTACTANNNGAATC
CGAACTCGGTACCAAGCTTGGCGNATCAAGGGGCATAACTTNNTTCCNGG

PRDN7-4

PRNP EGFP

Cas9-PRNP sgRNA-short vector/PRDN7-4
>131211-18_I11_PRDN7-4-T7promoter.ab1 1518
NNNGTGGCCTNNAATGATGCTCGAGCGGCCGCCAGTGTGATGGATATCTG
CAGAATTCGGCTTGCTCAAAGCTGTGGCAAACCCACCAGGTGTTTATTGT
TTTTTCCAGTTTAGATACAATGTATCAAGTAGAGGTTATTTTTACCATAA
GCATGTTGCTGGCATTCCACCTTTATCTTTTCTAAGAAACAGAGCCAGAA

AATTATCTGAAGGTCAAATTTGTCCTTAGAGAAGGAGAAAGAGTTAACCC
TTCACCTACAGTTGTTTTTGTTGTAAGTGTTGCACAGGAGACAAATGGAG
TATAAAGAACATTCACAGCTGATGCCACTACTATGTTCATTATGCTGCAG
ACATTAAGTGATTTCAATATAAACAGGACACTGACACCCTCTTTATTTTG
TATTTTGCAGATAAGTAATCATGGTGAAAAGCCATATAGGTGGCTGGATC
CTCGTTCTCTTTGTGGCCGCATGGAGTGACATAGGGCTCTGCAAGAAGCG
ACCAAAGCCTGGCGGAGGATGGAACACTGGGGGGAGCCGATACCCAGGGC
AGGGTAGTCCTGGAGGCAACCGCTATCCACCCCAGGGAGGGGGTGGCTGG
GGACAGCCCCACGGAGGTGGCTGGGGACAGCCCCACGGAGGCGGCTGGGG
ACAGCCCCACGGTGGCGGCTGGGGACAGCCCCATGGTGGCGGAGGCTGGG
GTCAAGGTGGTGGCTCCCACGGTCAGTGGAACAAGCCCAGTAAGCCGAAA
ACCAACATGAAGCATGTGGCAGGTGCCGCTGCAGCTGGGGCAGTGGTAGG
CGTACGTTAAAGATAATCATGCGTAAAATTGACGCATGTGTTTTATCGGT
CTGTATATCGAGGTTTATTTATTAATTTGAATAGATATTAAGTTTTATTA
TATTTACACTTACATACTAATAATAAATTCAACAAACAATTTATTTATGT
TTATTTATTTATTAAAAAAAAACAAAAACTCAAAATTTCTTCTATAAAGT
AACAAAACTTTTAACTAGTATGTCGAGGGACCTAATAACTTCGTATAGCA
TACATTATACGAAGTTATATTAAGGGTTCCGCAAGCTCTAGTCGAGCCCC
AGCTGGAAGCCGAATTCCAGCACACTGGCGGGCCGTTACTAATTGGATCC
AAGCTCGGTACCAAGCTTGGCGTAATCATGGTCNNNACTGTTTCCTGGGG

Supplementary Figure 5D. Control injections of GFP targeting vector only and Cas9:GFP only injections into activated porcine embryos. As seen below, residual expression of GFP was identified in targeting vector only injections. However, no targeting of the PRNP locus was identified as was expected. The residual expression could be a result of random integration into the genome, or persistence of transgene episomally within the embryo.

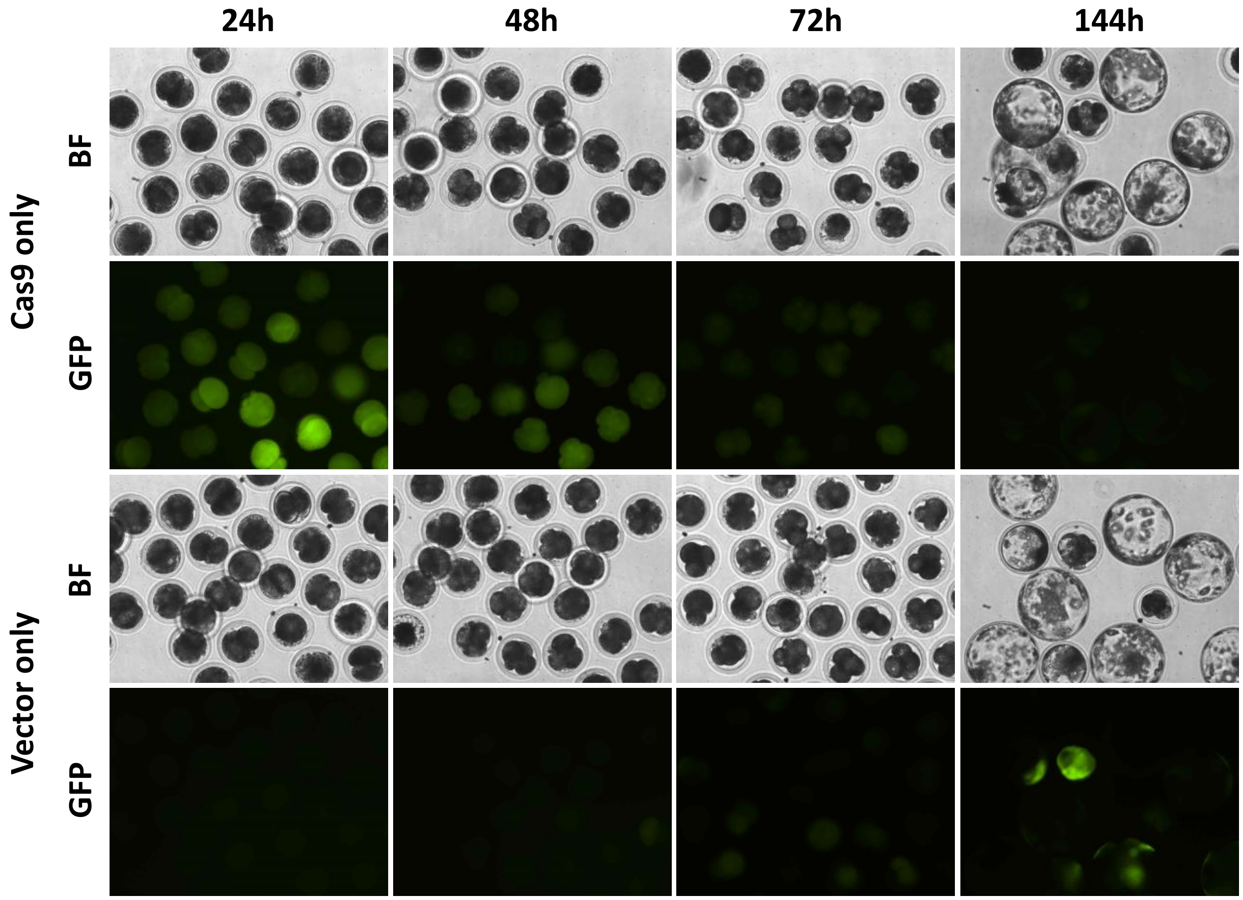

Amplification of micro injected blastocyst (Cas9 only and Vector only)

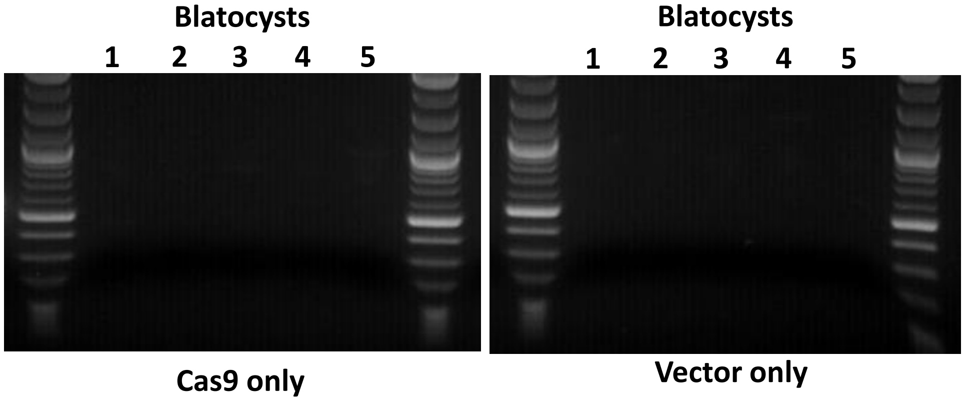

Supplementary Fig. S6. Two respective pregnancies from knock-in of pseudo attP sites into the *COL1A* locus were terminated on D60 of pregnancy and are shown below.

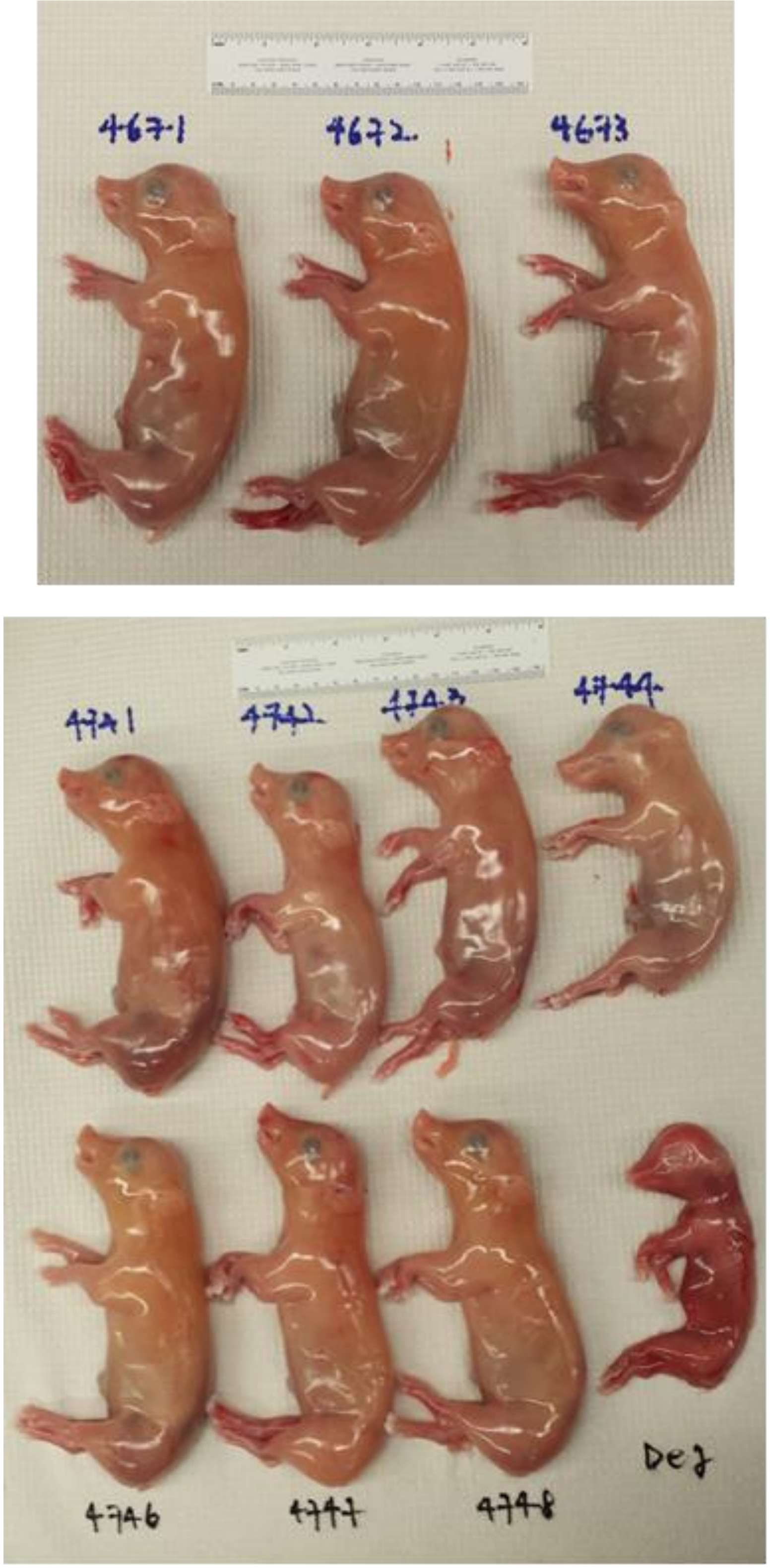

Supplementary Fig. S7. Genotyping of the fetuses from the pregnancies terminated on D60 of pregnancy. In the figure, fetus # 72, 41,46 and 47 are shown to carry a heterozygous insertion, 48 a homozygous insertion and 73 and 42 deletions. Fetus# 45 is degenerated and has a heterozygous insertion (weak bands not readily visible on the gel).

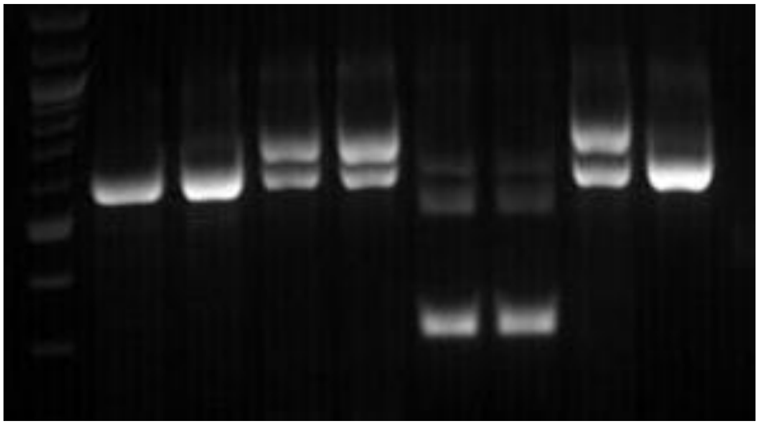

T E T E T E C WT

Fetus # 71 72 73

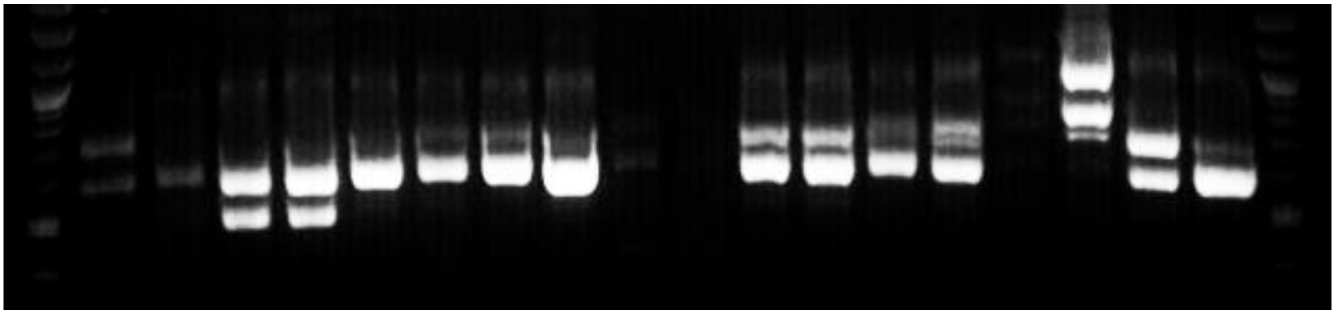

T E T E T E T E T E T E T E T E C WT

Fetus # 41 42 43 44 45 46 47 48

T: tail tissue C: target control
E: ear tissue WT: wild type

#45: Degenerated fetus

Supplementary Fig. S8. Investigation for incidence of mosaicism in gene targeted live offspring

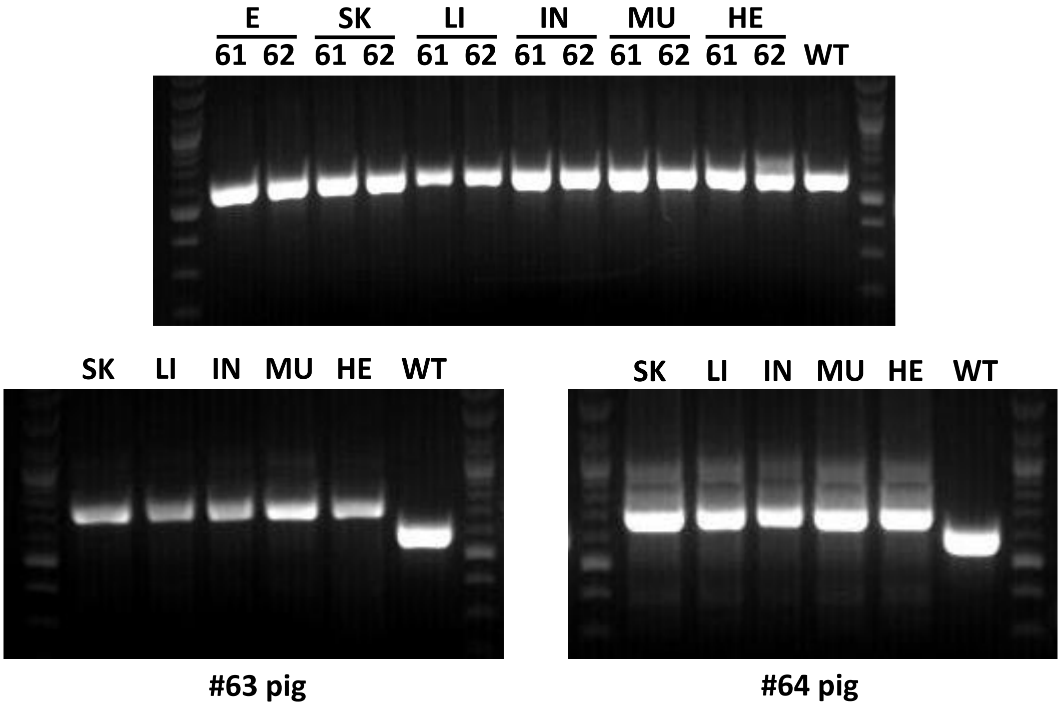

E: Ear

SK: Skin

LI: Liver

MU: Muscle

HE: Heart

IN: Intestine

WT: Wild type

Supplementary Fig. S9. Screening for off-targeting mutations in knock-in piglets. Top two genomic and non-genomic off-target genes were PCR amplified and sequenced. As shown below, no off-targeting events were identified in the candidates.

1st off target

```
COL1A sgRNA       --------TATCGAGGCTGGTCTCCCT-------------
Off target WT     TCCCCCTCTATTGAGGCTGGACTCCCTAGGATTCCTGCTC
Piglet #60        TCCCCCTCTATTGAGGCTGGACTCCCTAGGATTCCTGCTC
Piglet #61        TCCCCCTCTATTGAGGCTGGACTCCCTAGGATTCCTGCTC
Piglet #62        TCCCCCTCTATTGAGGCTGGACTCCCTAGGATTCCTGCTC
Piglet #63        TCCCCCTCTATTGAGGCTGGACTCCCTAGGATTCCTGCTC
Piglet #64        TCCCCCTCTATTGAGGCTGGACTCCCTAGGATTCCTGCTC
```

2nd off target

```
COL1A sgRNA       ------------TATCGAGGCTGGTCTCCCT----------
Off target WT     CCCAATCAGCTCTTTCGAGGCTGGGCTCCCTGGCAGTCTTC
Piglet #60        CCCAATCAGCTCTTTCGAGGCTGGGCTCCCTGGCAGTCTTC
Piglet #61        CCCAATCAGCTCTTTCGAGGCTGGGCTCCCTGGCAGTCTTC
Piglet #62        CCCAATCAGCTCTTTCGAGGCTGGGCTCCCTGGCAGTCTTC
Piglet #63        CCCAATCAGCTCTTTCGAGGCTGGGCTCCCTGGCAGTCTTC
Piglet #64        CCCAATCAGCTCTTTCGAGGCTGGGCTCCCTGGCAGTCTTC
```

3rd off target

```
COL1A sgRNA       ------------TATCGAGGCTGGTCTCCCT----------
Offtarget WT      TTCATCTCACTTTATTGGGGCTGGTGTCCCTTTGGGCTCGA
Piglet #60        TTCATCTCACTTTATTGGGGCTGGTGTCCCTTTGGGCTCGA
Piglet #61        TTCATCTCACTTTATTGGGGCTGGTGTCCCTTTGGGCTCGA
Piglet #62        TTCATCTCACTTTATTGGGGCTGGTGTCCCTTTGGGCTCGA
Piglet #63        TTCATCTCACTTTATTGGGGCTGGTGTCCCTTTGGGCTCGA
Piglet #64        TTCATCTCACTTTATTGGGGCTGGTGTCCCTTTGGGCTCGA
```

4th off target

```
COL1A sgRNA      ---------TATCGAGGC-TGGTCTCCCT------------
Off target WT    GCCCCCGCGGAGCAGGGCACAGACTCCCTGGGCGTGTGGCA
Piglet #60       GCCCCCGCGGAGCAGGGCACAGACTCCCTGGGCGTGTGGCA
Piglet #61       GCCCCCGCGGAGCAGGGCACAGACTCCCTGGGCGTGTGGCA
Piglet #62       GCCCCCGCGGAGCAGGGCACAGACTCCCTGGGCGTGTGGCA
Piglet #63       GCCCCCGCGGAGCAGGGCACAGACTCCCTGGGCGTGTGGCA
Piglet #64       GCCCCCGCGGAGCAGGGCACAGACTCCCTGGGCGTGTGGCA
```

Supplementary Fig.S10: Targeted knockin of GFP expression cassette into the pseudo attP sites introduced downstream of *COL1A* site. A) The GFP donor plasmid and phiC31 Integrase expression plasmids. B) The expression plasmids were nucleofected into *COL1A* attP knock-in porcine fetal fibroblasts. The nucleofected cells were selected with G418 for two weeks and the cells were flow sorted. C) The cells that survived G418 selection, and sorted based on GFP were screened for knock-in of transgenes. Only the cells nucleofected with GFP donor and integrase plasmids tested positive for transgene knock-in and not the cells nucleofected with GFP transgene alone confirming functionality of the integrated attP sites and integrase approach.

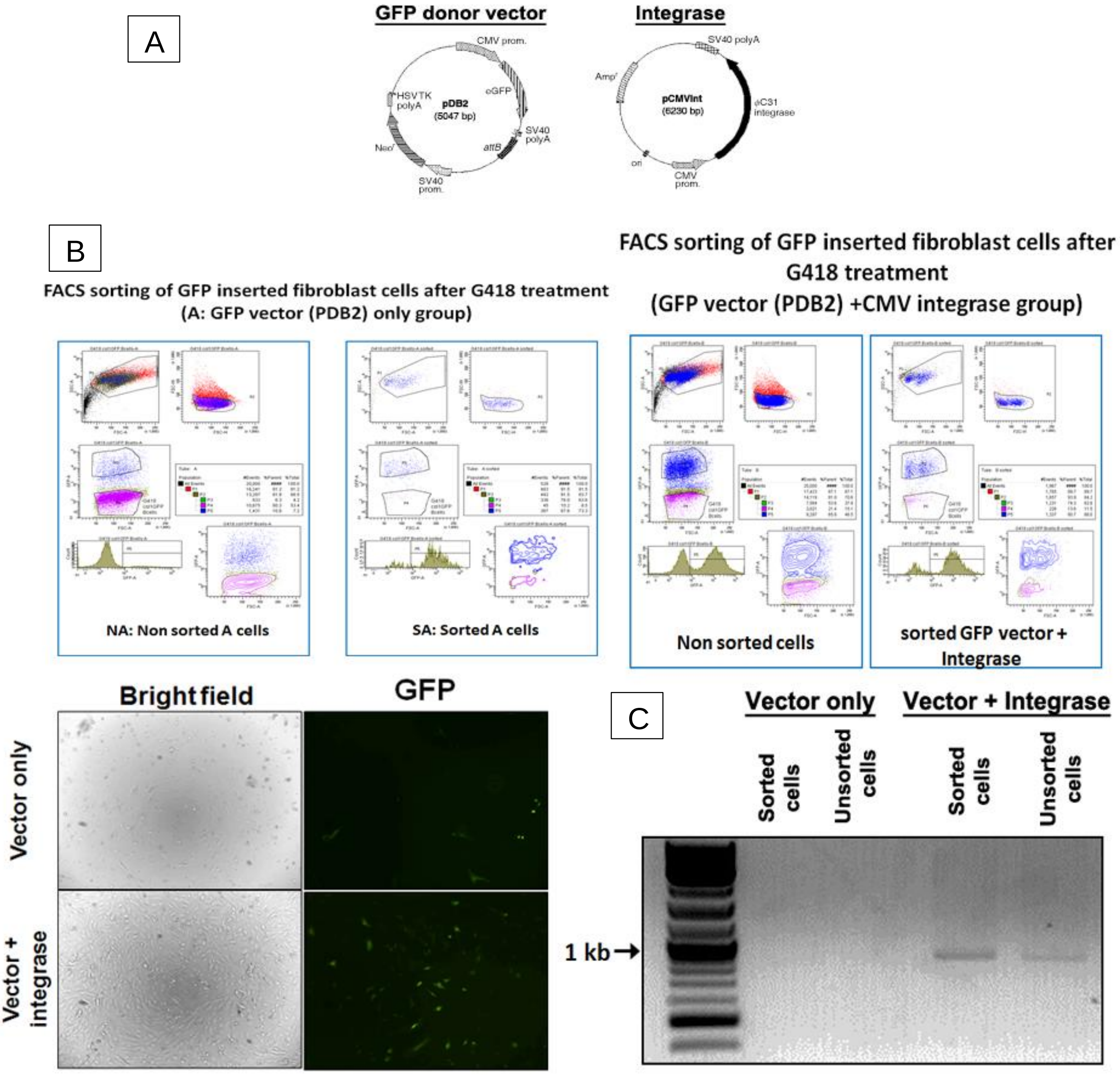

Supplementary Fig. S11: Sequences of primers used in the manuscript

Gene	Primer sequence (5'-3')	Fragment size (bp)	Gene Bank Accession #
ZBED6 For	TGCTGGGATTCTGGGCTGTG	423	NC_010451
ZBED6 Rev	GCTACCTGGTTTACCCCTGCT		
PRNP upper arm BsiW1 rev	GACTACGTACGCCTACCACTGCCCCAGCTG	1000	NC_010459
PRNP upper arm MluI for	CTAGACGCGTCTTGGCACAGGGGCCCTAG		
PRNP lower arm XhoI for	GACTCTCGAGCGGTTACATGCTGGGGAGT	1000	
PRNP lower arm AscI rev	GTCAGGCGCGCCTTGATGCAATAGCTGCTGT		
PRNP screen for	CCAGCTGGGGCTCGACTAGA	947	
PRNP screen rev 2	GCTCAAAGCTGTGGCAAACC		
col1a1 screen for2	AGCCAGGCTGCCTTGTTTG	579	NC_010454
col1a1 screen rev2	GCCAACCTCCCCTTTGCACT		
